# Rhenium-Selenido Corroles: Reflections on 5d Metalloporphyrins and Metallocorroles as Triplet Emitters and Photosensitizers

**DOI:** 10.1021/acs.inorgchem.5c01593

**Published:** 2025-06-09

**Authors:** Abraham B. Alemayehu, Jeanet Conradie, Simon Larsen, Bjørn Cicerôn Lukas Pérez, Nicholas S. Settineri, Abhik Ghosh

**Affiliations:** † Department of Chemistry, 8016 UiT − The Arctic University of Norway, Tromsø N-9037, Norway; ‡ Department of Chemistry, University of the Free State, P.O. Box 339, Bloemfontein 9300, Republic of South Africa; § Advanced Light Source, 1438 Lawrence Berkeley National Laboratory, Berkeley, California 94720-8229, United States

## Abstract

Following the successful synthesis of rhenium-oxido, rhenium-imido, and rhenium-sulfido corroles, a series of *para*-X-substituted rhenium-selenido triarylcorroles (X = OCH_3_, CH_3_, H, F, CF_3_) have been prepared in 70–76% yields. A one-pot, two-step procedure was used, analogous to that used for ReS corroles, involving rhenium insertion via high-temperature reaction of Re_2_(CO)_10_ and free-base corroles in refluxing decalin, followed by exposure to PCl_3_ (which is thought to deoxygenate ReO corroles formed under the reaction) and elemental selenium. An analogous procedure, however, failed to yield rhenium-tellurido corroles, presumably reflecting, according to DFT calculations, the limited stability of these species. Unlike ReO corroles, ReS and ReSe corroles were found not to exhibit phosphorescence in the NIR-I regime (600–1000 nm); nor did they sensitize singlet oxygen formation. In the hope of obtaining a broader perspective of this negative result, we also examined ReN porphyrins and found them not to phosphoresce or to sensitize singlet oxygen formation. These results were explained by DFT and TDDFT calculations in terms of low-energy triplet states, which are not energetic enough to excite molecular oxygen to its lowest singlet state. Whether some of the new Re corroles exhibit phosphorescence in the NIR-II regime remains an interesting question for future investigation .

## Graphical abstract

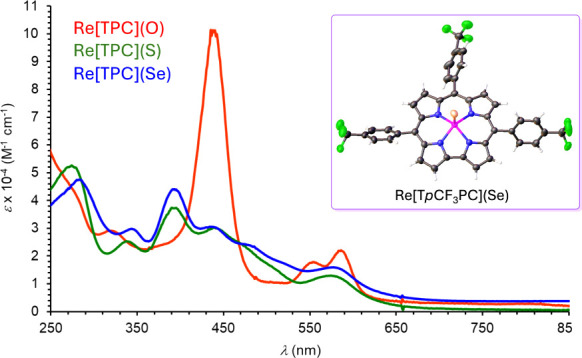

## Introduction^1^

The interactions of porphyrin-type ligands and rhenium have been studied for over a half-century.^2,3^ In a striking, early discovery, Tsutsui and coworkers reported that porphyrins can act as binucleating ligands toward rhenium, yielding binuclear [Por]­[Re­(CO)_3_]_2_ complexes, in which the two rhenium atoms straddle opposite faces of the porphyrin, with each metal coordinated to three porphyrin nitrogens and two of the porphyrin nitrogens coordinated to both metals.^4,5^ Key examples of other coordination motifs involving porphyrins include six-coordinate rhenium-oxido and five-coordinate rhenium-nitrido porphyrins.^6^ A striking rhenium-porphyrin interaction was reported in 1998, in which attempted rhenium insertion into *meso*-tetrakis­(trifluoromethyl)­porphyrin resulted in an unexpected ring contraction affording Re^V^O *meso*-tris­(trifluoromethyl)­corrole.^7^ The latter compound was the first example of a corrole derivative of a 5d transition metal; today, many such complexes are known^8–17^ and recognized for their luminescence properties and potential application in photomedicine.^18–23^ Subsequently, with the availability of one-pot corrole syntheses,^24^ Re^V^O corroles could be synthesized simply and in high yields via the interaction with free-base *meso*-triarylcorroles and Re_2_(CO)_10_ in a high-boiling solvent.^25–28^ Tinkering with the reaction conditions (especially the temperature) led to additional coordination motifs, including metal–metal quadruple-bonded rhenium corrole dimers^29,30^ and rhenium biscorrole sandwich compounds.^31^ Still other coordination motifs were obtained by the introduction of additional reagents into the reaction medium; key examples include rhenium-imido^32^ and rhenium-sulfido corroles.^33^ In this study, we investigated the effect of selenium and tellurium-based additives and the successful synthesis and characterization of rhenium-selenido corroles. The latter constitute rare examples of stable, structurally characterized terminal rhenium-selenido complexes.

The availability of a wide range of rhenium­(V) porphyrin and corrole derivatives encouraged us to undertake a comparative photophysical study of ReO,^24–27^ Re-imido,^31^ ReS,^32^ and ReSe corroles and ReN porphyrins.^34^ Unfortunately, unlike ReO corroles (which exhibit near-infrared phosphorescence at room temperature, singlet oxygen sensitization, and photocytotoxicity toward multiple cell lines,^35,36^ ReS and ReSe corroles and ReN porphyrins proved nonemissive in the 600–1000 nm range and also incapable of sensitizing singlet oxygen formation. A ground state and time-dependent density functional theory (DFT and TDDFT) analysis of a selection of metalloporphyrins and metallocorroles, however, yielded deep insights into their widely varying photophysical behavior. These insights are likely to be a valuable aid in the future design of new triplet emitters and photosensitizers (Chart [Fig chart1]).

**1 chart1:**
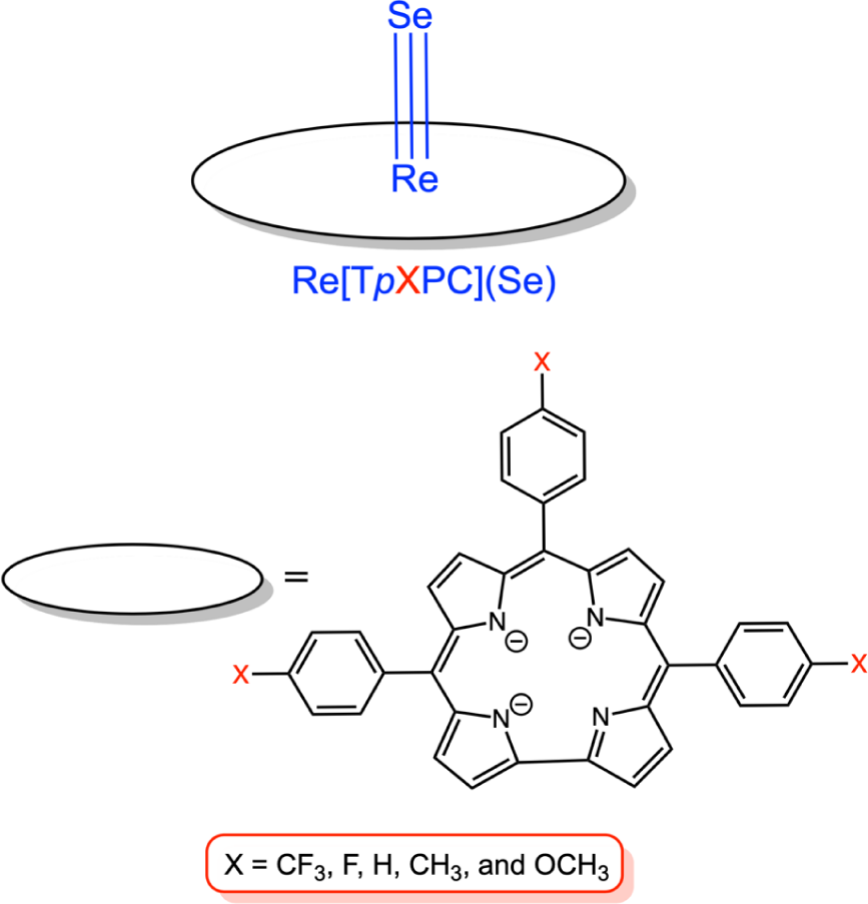
Complexes Synthesized As Part of This Work

## Results and Discussion

### Discovery and Optimization of Synthetic Methods

In a recent paper reporting the synthesis of stable rhenium-sulfido corroles,^32^ we presented DFT-based thermochemical arguments in favor of similarly stable rhenium-selenido corroles. That prediction has now been realized via a simple, one-pot, two-step synthesis of ReSe corroles. Rhenium insertion was first accomplished (typically an overnight process) via the high-temperature interaction of a free-base *meso*-**t**ris­(***p***-**X**-**p**henyl)**c**orrole, H_3_[T*p*XPC] (X = OCH_3_, CH_3_, H, F, CF_3_; **Chart 1**), Re_2_(CO)_10_, and potassium carbonate in refluxing decalin (∼180 °C). In the next step, phosphorus trichloride and elemental selenium powder was introduced into the reaction mixture, and the reaction was continued for another ∼ 4 h, at the end of which Re­[T*p*XPC]­(Se) complexes were isolated as air-stable solids in yields of 70.0–75.4%.

High-resolution mass spectra, ^1^H NMR spectroscopy (Figure 1) and three single-crystal X-ray diffraction analyses (Table 1 and Figure 2) provided unambiguous proof of composition and structure of the new compounds. To freeze out interconversion of the *meso*-aryl *o*,*ó* and *m*,*ḿ* peaks, fully assigned ^1^H NMR spectra were recorded at 243 K, by now a standard practice for square-pyramidal metallocorroles. Given the rarity of terminal rhenium-selenido complexes (in contrast to molybdenum- and tungsten-selenido complexes, which are well-represented in the literature and in the Cambridge Structural Database), the Re–Se distances were of considerable interest for us. The observed distances, 2.1881(5)-2.2157(5) Å, are slightly shorter than the value, 2.2668(9) Å, obtained for a tetrahedral Re­[β-diketiminato]­(NPh)­(Se) complex.^37^ Interestingly, the Re–Se distances observed for our complexes are about halfway between the sums of Pyykkö’s covalent radii for double and triple bonds. (The double and triple bond radii for Re are 1.19 and 1.10 Å, respectively; for Se, the values are, each, 1.07 Å.^38,39^ The observation suggests that the Re–Se bond has substantial, but not quite full, triple bond character. Natural bond orbital (NBO) analyses of ReCh corroles (Ch = O, S, Se, i.e., a chalcogen) lend support to such a conclusion. As shown in Figure 3, while the occupancies of Re-Ch π -NBOs are roughly independent of the chalcogen, the corresponding π* occupancies are significantly higher for the heavier chalcogens (relative to oxygen), consistent with a lower overall bond order. Other aspects of the structures, such as the Re–N distances (involving the corrole nitrogens) and the displacement of the Re from mean plane of the corrole nitrogens, are relatively unremarkable and do not appear to merit comment.

**Figure 1:**
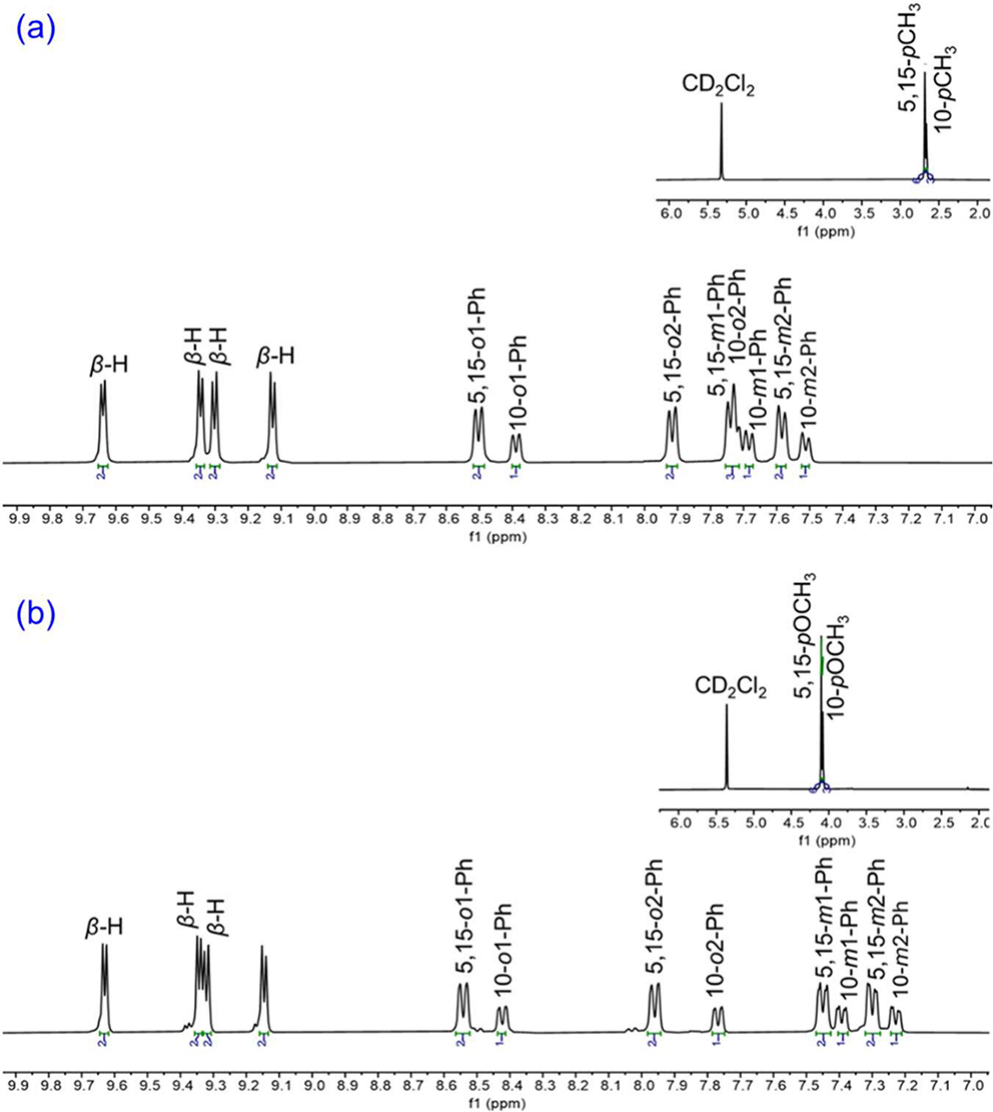
^1^H NMR spectra of (a) Re­[T*p*CH_3_PC]­(Se) and (b) Re­[T*p*OCH_3_PC]­(Se) in CD_2_Cl_2_ at 243 K.

**Table 1:** Crystal Data and Structure Refinement Parameters

Compound	Re[T*p*OCH_3_PC](Se)	Re[T*p*CH_3_PC](Se)	Re[T*p*CF_3_PC](Se)
CCDC deposition number	2435739	2435738	2435737
Method	SC-XRD	SC-XRD	SC-XRD
Chemical formula	C_40_ H_29_ N_4_ O_3_Se Re	C_40_ H_29_ N_4_ Se Re	C_40_ H_20_ F_9_ N_4_ Se Re
Formula weight	878.83	830.83	992.76
Crystal system	Orthorhombic	Triclinic	Monoclinic
Crystal dimensions	0.060 × 0.060 × 0.050	0.080 × 0.040 × 0.010	0.060 × 0.020 × 0.010
Space group	*Pccn*	P-1	*P*21/c
λ (Å)	0.7288	0.7288	0.7288
*a* (Å)	26.194(2)	13.1157(7)	16.1470(12)
*b* (Å)	15.2424(13)	15.9836(8)	15.2888(11)
*c* (Å)	16.4190(13)	16.7850(9)	14.0172(10)
α (°)	90	66.324(3)	90
β(°)	90	87.379(3)	94.796(3)
γ (°)	90	80.778(3)	90
*Z*	8	4	4
*V* (Å^3^)	6555.6(10)	3180.2(3)	3448.3(4)
Temperature (K)	100(2)	100(2)	100(2)
Density (g/cm^3^)	1.781	1.735	1.912
Measured reflections	186678	120210	99985
Unique reflections	8218	24423	8632
Parameters	445	835	537
Restraints	6	0	87
*R*_int_	0.0590	0.0524	0.0683
θ range (°)	1.585 – 29.205	1.359 – 34.301	1.298 – 29.190
*R*_1_ [*I* ≥ 2σ(*I*)], *wR*_2_ [all data]	0.0627, 0.1582	0.0551, 0.0964	0.0449, 0.0954
*S* (GooF) all data	1.073	1.056	1.057
Max/min residue (e/Å^3^)	3.655 /-2.032	4.301 /-5.454	1.339/-1.751

**Figure 2:**
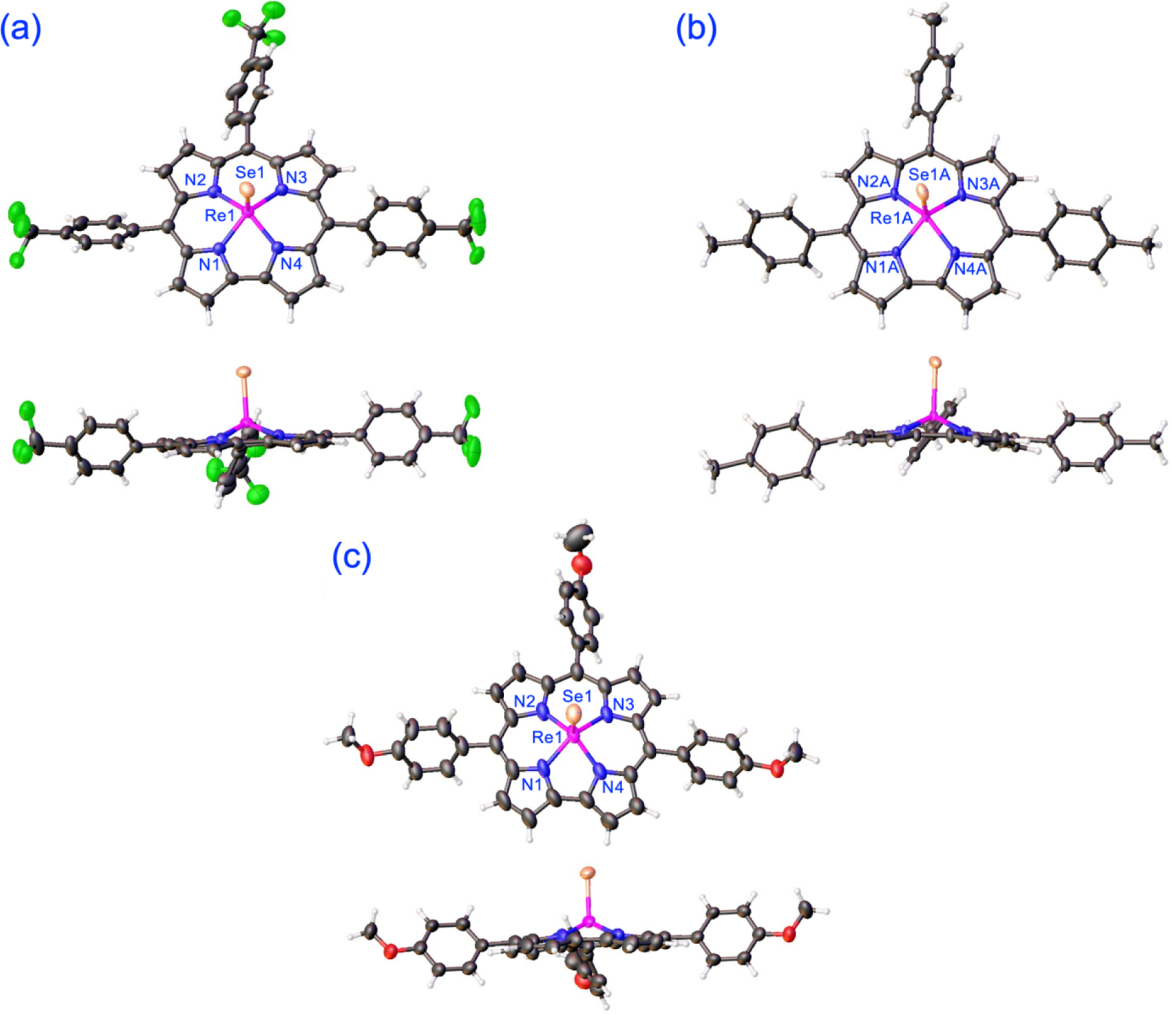
X-ray structures (top and side views) of Re­[T*p*XPC]Se complexes. Selected distances (Å): **(a) Re­[T** *p* **CF**_**3**_ **PC]­(Se).** Re1–N1 1.985(4), Re1–N2 2.011(3), Re1–N3 2.023(3), Re1–N4 1.984(4), Re1–Se1 2.1881(5). **(b) Re­[T** *p* **CH**_**3**_ **PC]­(Se).** Re1A-N1A 1.979(3), Re1A-N2A 2.007(3), Re1A-N3A 1.998(3), Re1A-N4A 2.000(4), Re1A-Se1A 2.2157(5); 1.998(3), Re1A-N4A 2.000(4), Re1A-Se1A 2.2157(5); Re1B–N1B 1.983(3), Re1B–N2B 2.009(3), Re1B–N3B 2.002(3), Re1B–N4B 1.983(3), Re1B–Se1B 2.2094(5). **(c) Re­[T** *p* **OCH**_**3**_ **PC]­(Se).** Re1–N1 2.007(5), Re1–N2 1.986(5), Re1–N3 2.003(5), Re1–N4 2.006(6), Re1–Se1 2.2101(7). The thermal ellipsoids correspond to 50% probability. Positional disorder and solvent molecules have been omitted for clarity.

**Figure 3:**
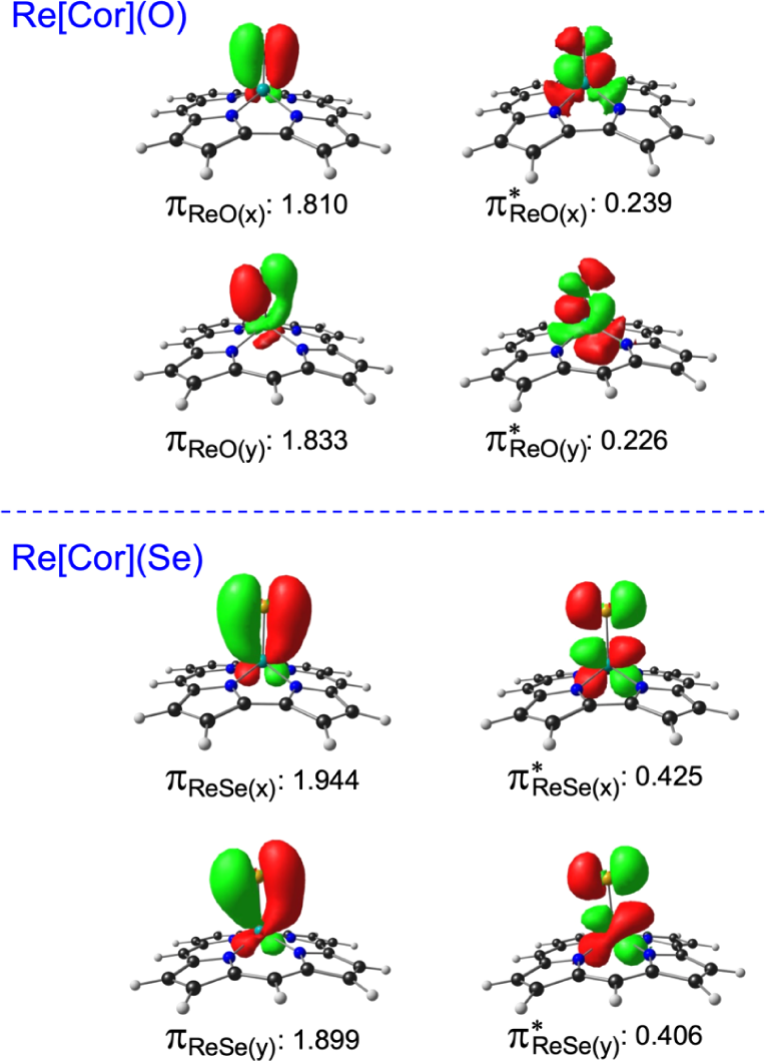
Re-Ch (Ch = O, Se) π and π * NBO occupancies based on OLYP-D3/ZORA-STO-TZ2P calculations. (Note that the x direction is parallel to the direct pyrrole–pyrrole linkage.).

It may be of some interest to view the present syntheses through a geochemical lens.^40^ Although inorganic chemists often think of rhenium as an oxophilic element,^41–43^ rhenium occurs as a trace constituent of both oxide minerals such as columbite, (Fe,Mn)­(Ta,Nb)_2_O_6_, and the sulfide mineral molybdenite, MoS_2_. Indeed a recently developed, quantitative scale of thiophilicity (based on element-chalcogen bond energies) ranks rhenium as equally oxophilic and thiophilic.^44^ The successful synthesis of stable ReO,^24–27^ ReS,^32^ and ReSe corroles appears eminently consonant with such a view.

### Electrochemical, Optical, and Photophysical Studies

Cyclic voltammetry, UV–vis absorption spectroscopy and photophysical studies yielded a fascinating system of electronic-structural insights (Table 2). On the whole, the results closely parallel those obtained for ReS corroles.

**Table 2:** Electronic Absorption Maxima (*λ*_max_, Nm) and Redox Potentials (V vs SCE) of Re­[T*p*XPC]­(Y), for Y = Se, S, O, and NPh

Compound	λ_max_	*E*_1/2(ox2)_	*E*_1/2(ox1)_	*E*_1/2(red1)_	*E*_1/2(red2)_	Δ*E*	ref
Re[T*p*CF_3_PC](Se)	280, 344, 393, 471, 577	1.58	1.14	–1.16	–1.69	2.30	This work
Re[T*p*FPC](Se)	280, 344, 391, 471, 574	1.45	0.98	–1.17	–1.70	2.15	
Re[TPC](Se)	282, 345, 393, 430, 577	1.44	0.95	–1.20	–1.74	2.15	
Re[T*p*CH_3_PC](Se)	280, 343, 393, 438, 577	1.43	0.94	–1.21	–1.77	2.15	
Re[T*p*OCH_3_PC](Se)	283, 346, 393, 439, 578	1.32	0.91	–1.23	–1.79	2.14	
Re[T*p*CF_3_PC](S)	276, 340, 393, 459, 571	1.59	1.07	–1.15	–1.68	2.22	
Re[T*p*FPC](S)	276, 341, 391, 459, 571	1.54	1.00	–1.22	–1.79	2.22	
Re[TPC](S)	277, 339, 391, 459, 572	1.52	0.96	–1.23	–1.80	2.19	
Re[T*p*CH_3_PC](S)	276, 339, 392, 459, 575	1.46	0.92	–1.25	–1.84	2.17	
Re[T*p*OCH_3_PC](S)	273, 344, 394, 459, 574	1.35	0.89	–1.27	–1.85	2.16	
Re[T*p*CF_3_PC](O)	438, 585	-	1.10	–1.16	-	2.26	24
Re[T*p*FPC](O)	438, 585	-	1.01	–1.23	-	2.24	
Re[TPC](O)	439, 585	-	0.98	–1.26	-	2.24	
Re[T*p*CH_3_PC](O)	440, 587	-	0.94	–1.29	-	2.23	
Re[T*p*OCH_3_PC](O)	441, 592	-	0.93	–1.29	-	2.22	
Re[T*p*CF_3_PC](NPh)	434, 577	1.24	0.97	–1.29	-	2.26	31
Re[T*p*FPC](NPh)	434, 575	1.15	0.88	–1.36	-	2.24	
Re[TPC](NPh)	434, 576	1.18	0.86	–1.38	-	2.24	
Re[T*p*CH_3_PC](NPh)	434, 578	1.12	0.82	–1.40	-	2.22	
Re[T*p*OCH_3_PC](NPh)	435, 578	1.03	0.78	–1.41	-	2.19	

Cyclic voltammetry revealed two reversible oxidations and at least one reversible reduction (as well as second quasi-reversible reduction) for each ReSe corrole (Figure 4). The complexes were found to exhibit relatively high first oxidation potentials, 0.89 to 1.07 V, and relatively low first reduction potentials, −1.27 to −1.15 V, vs the SCE, as might be expected for an electronically innocent corrole macrocycle with a high-valent central metal ion. The electrochemical HOMO–LUMO gap (i.e., the algebraic difference between the first oxidation and reduction potentials) of 2.2 V is also essentially the same as that observed for ReO,^24^ ReS,^32^ OsN,^9^ Au^15,27^ and other electronically innocent metallocorroles. This HOMO–LUMO gap is also consistent with that inferred from the lowest-energy optical absorption maxima of the compounds (Table 2 and Figure 5). These observations might be naively interpreted as indicative of a purely ligand-based HOMO and a purely ligand-based LUMO, as expected from Gouterman’s four-orbital model.^45–48^ [According to this model, the two π HOMOs of a porphyrin are near-degenerate, as are the two LUMOs, and these four MOs are energetically well-separated from all other occupied and unoccupied MOs; although originally formulated for porphyrins, the model has been found to hold for many simple corroles such as Ga and Au corroles.^49,50^ We shall see that such a conclusion would be entirely fallacious: the valence electronic structures of ReS and ReSe corroles are utterly at odds with the four-orbital model. Indeed, the broad, smeared-out features in the 370–650 nm region of the optical spectra of ReS and ReSe corroles already hint at a distinctly non-Gouterman-type MO architecture.

**Figure 4:**
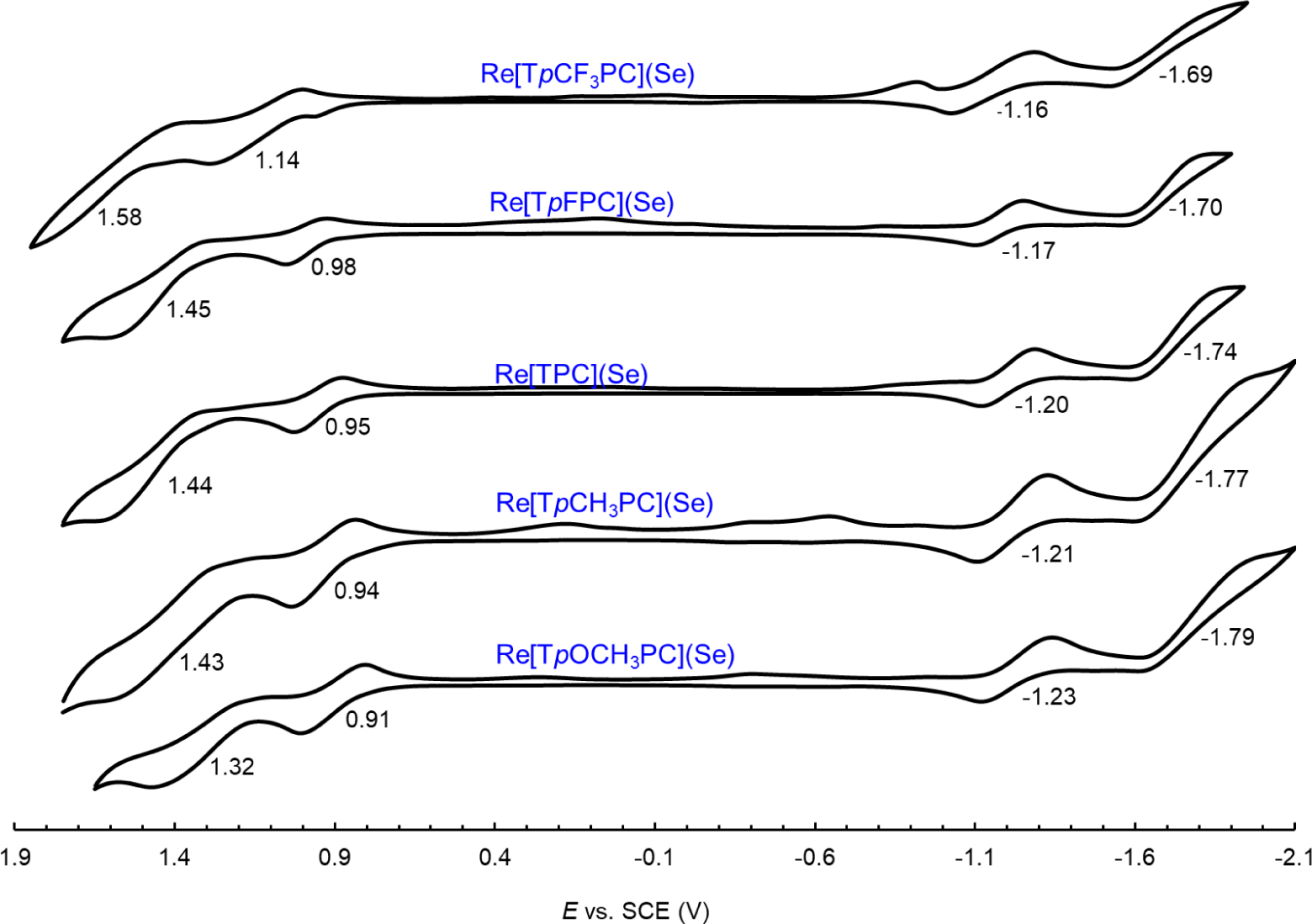
Cyclic voltammograms of Re­[T*p*XPC]­(Se) in 0.1 M solutions of tetrabutylammonium perchlorate in anhydrous dichloromethane; scan rate = 100 mV.s^–1^.

**Figure 5:**
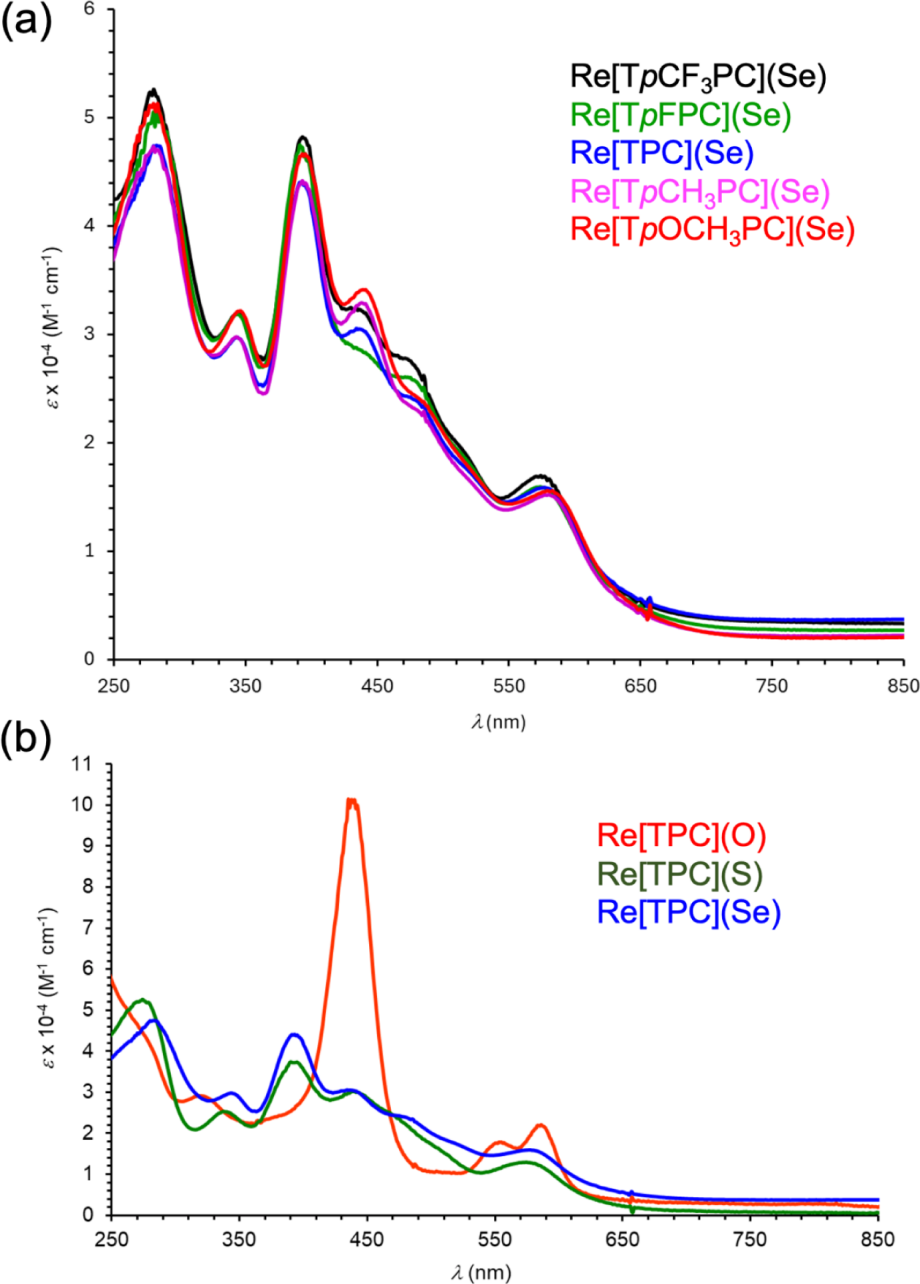
UV–vis spectra in dichloromethane: (a) the Re­[T*p*XPC]­(Se) series; (b) Re­[TPC]­(Ch) (Ch = O, S, Se). Sample concentrations were in the range 4.0 ± 0.5 mM. See Table 2 for a listing of peak maxima.

Additional electronic-structural clues came from photophysical studies. Since ReO corroles were previously found to exhibit moderate NIR phosphorescence and to efficiently generate singlet oxygen,^34,35^ the potential emissive properties of the ReS and ReSe corroles were investigated. Somewhat disappointingly, both higher- and lower-energy excitation of their toluene solutions failed to elicit any emission in the 600–1000 nm range. Since emission properties (particularly phosphorescence) are often significantly enhanced at low temperatures, the measurements were repeated at 77K in toluene:tetrahydrofuran (2:3 v/v) frozen glass, but to no avail. Finally, we tested both ReS and ReSe corroles for singlet oxygen sensitization in ethanol using 9,10-dimethylanthracene as a trap (with excitation at 570 nm). None was observed.

Given that many iridium porphyrins exhibit intense phosphorescence,^51–53^ relative to iridium corroles, which are only weakly phosphorescent,^54–56^ we chose to examine rhenium-nitrido porphyrins.^33^ Several were prepared according to literature procedures, but only two, based on electron rich *meso*-tetrakis­(*p*-X-phenyl)­porphyrins (X = Me, OMe) proved stable enough for photophysical studies. A number of Re­(O)­(Cl) porphyrins were also prepared, but these too proved rather unstable, precluding reliable photophysical measurements.^1,2^ Disappointingly, unlike their Ir counterparts, the ReN porphyrins did not exhibit phosphorescence (in the 600–1000 nm range) or singlet oxygen sensitization.

### DFT and TDDFT Calculations

To make sense of the wide range of photophysical properties of rhenium porphyrins and corroles, and of 5d metalloporphyrins^50–52,57–62^ and metallocorroles^18–21,27,53–55,63–68^ in general, we undertook a DFT and TDDFT survey of over a dozen complexes. The B3LYP*^69^ Kohn–Sham MO energy level diagrams (Table 3, Figures 6, and S11–S13) alone provided certain insights into why phosphorescence and singlet-oxygen sensitization are observed for certain species, but not for others. Thus, an unusually low HOMO–LUMO gap was found for unsubstituted ReN porphyrin, suggesting that the necessarily low-energy triplet state would not phosphoresce in our spectral window (600–1000 nm) or excite molecular oxygen to its lowest singlet state (which has an energy of 1.2 eV above the triplet ground state). Second, the MO architecture of ReS and ReSe corrole exhibits little resemblance to Gouterman’s four-orbital model; the LUMOs of these complexes are substantially metal-based and profoundly different from those of ReO corrole (Figure 7), which qualitatively accounts for the dramatic differences in UV–vis spectral profile among different Re-chalcogenido corroles (Figure 5b).

**Figure 6:**
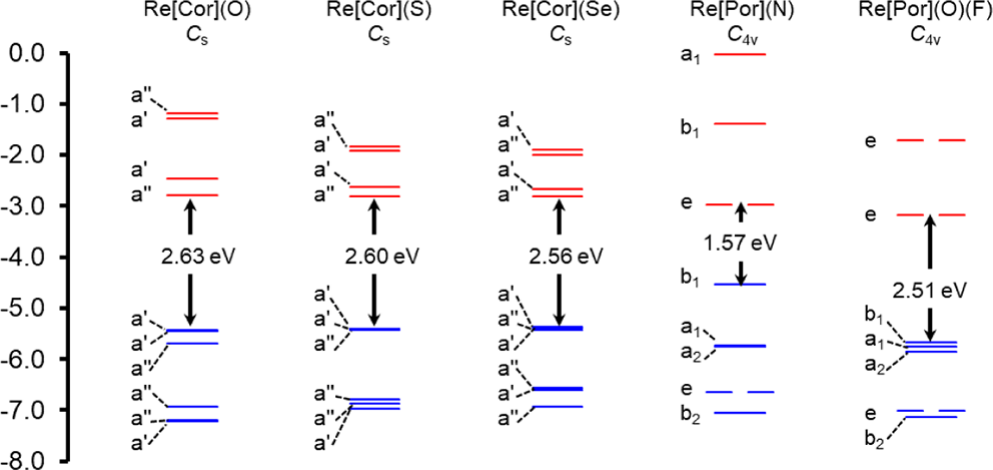
B3LYP*/STO-ZORA-TZ2P MO energy level diagrams of Re porphyrins and corroles based on OLYP-D3 ground-state optimized geometries. Cor and Por refer to unsubstituted corrolato­(3-) and porphyrinato­(2-) ligands, respectively. Doubly occupied and unoccupied MO energy levels are indicated in blue and red, respectively.

**Table 3:** Calculated Triplet Energies (eV) from Regular Scalar-Relativistic (SR) and Time-Dependent (TD) Scalar- (SR) and Spin-Orbit (SO) Relativistic B3LYP* Calculations

	SR-B3LYP*	TD-SR-B3LYP*^b^		TD-SO-B3LYP*^b^ ^c^	
	*E*_T1_ ^a^	*E*_T1_	*E*_S1_	Lowest excitation energy	*f*
*Porphyrins*					
Re[Por](N)	0.83	0.55	0.58	0.56	3.18 × 10^–8^
Re[Por](O)(F)	1.25	1.19	1.45	1.19	2.93 × 10^–7^
Ir[Por](Me)	1.64	1.55	1.77	1.46	1.02 × 10^–5^
Pd[Por]	1.84	1.79	2.46	1.79	1.43 × 10^–8^
Pt[Por]	1.93	1.89	2.35	1.89	2.35 × 10^–7^
*Corroles*					
Re[Cor](O)	1.58	1.54	2.06	1.54	6.90 × 10^–8^
Re[Cor](S)	0.98	0.98	1.38	0.99	5.69 × 10^–7^
Re[Cor](Se)	0.88	0.87	1.28	0.87	5.42 × 10^–7^
Ru[Cor](N)	1.50	1.46	1.82	1.46	3.63 × 10^–7^
Os[Cor](N)	1.56	1.52	2.28	1.52	1.60 × 10^–9^
Ir[Cor](py)_2_	1.51	1.47	2.10	1.47	1.64 × 10^–6^
Pt[Cor](Ph)(py)	1.51	1.47	2.25	1.47	1.90 × 10^–8^
Au[Cor]	1.57	1.52	2.42	1.52	1.46 × 10^–8^

a These triplet energies are adiabatic singlet–triplet gaps and were obtained from single point scalar-relativistic B3LYP* calculations on symmetry-unconstrained OLYP-D3 optimized geometries for *M*_*S*_ = 0 and 1.

b Both scalar-relativistic and spin–orbit time-dependent B3LYP* calculations were carried out on symmetry-unconstrained OLYP-D3 optimized geometries for *M*_*S*_ = 1. The lowest excitation energies thus correspond to phosphorescence from a geometry-relaxed triplet state.

c The spin–orbit relativistic oscillator strengths *f* are expected to be proportional to phosphorescence quantum yields.

**Figure 7:**
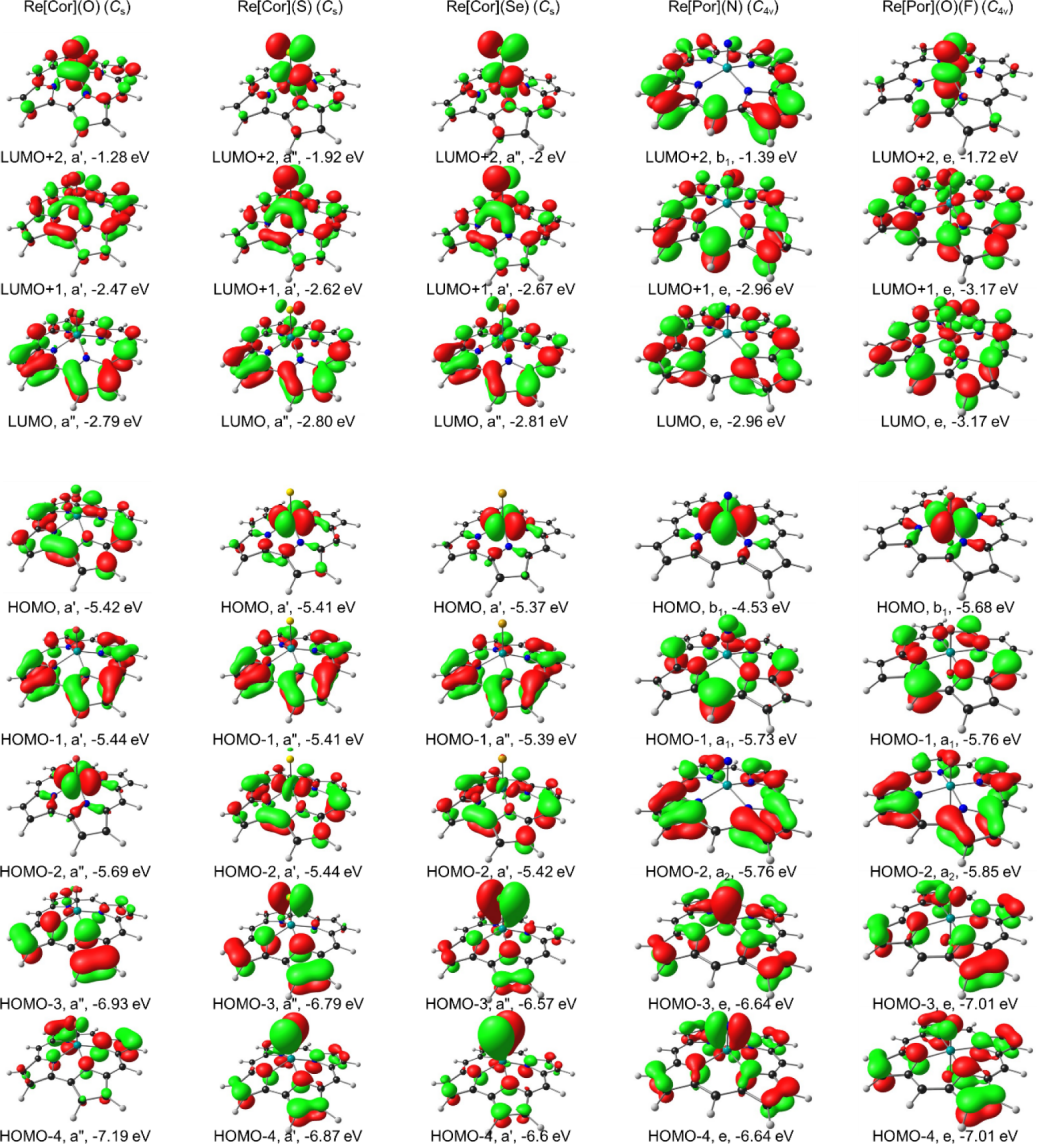
B3LYP*/STO-ZORA-TZ2P frontier MOs (with a surface isovalue of 0.05 e/Å^3^) of Re porphyrins and corroles based on OLYP-D3 ground-state optimized geometries.

To explain the observed photophysical behavior of a wider range of complexes (Table 3), we chose two computational approaches. In the first, ground-state DFT methods were used to calculate single-point “singlet” and “triplet” energies at the “triplet” geometry (obtained with scalar-relativistic OLYP-D3 calculations). Given the longer time scale of phosphorescence, the singlet–triplet gap thus obtained at a relaxed “triplet” geometry difference corresponds to the phosphorescence energy. (Note that we are using the expressions “singlet” and “triplet” here in a loose sense; more accurately, we carried out geometry optimizations for *M*_*S*_ = 0 and 1.) In the second approach, TDDFT calculations were carried out on singlet reference states with optimized triplet geometries. Again, the triplet energies thus obtained correspond to phosphorescence energies. Both scalar-relativistic and spin–orbit ZORA Hamiltonians were used in the TDDFT calculations. Reassuringly, the triplet state energetics obtained with the different methods (Table 3) proved largely mutually consistent. To our satisfaction, the results also went a long way toward rationalizing the observation or otherwise of phosphorescence and singlet oxygen sensitization by key species.

Table 3 indicates substantial HOMO–LUMO gaps for ReO,^34,35^ OsN,^62^ Ir,^53–55^ PtPh^63^ and Au^64–67^ corrole as well as for Pd, Pt,^56–61^ and IrMe^50–52^ porphyrin, consistent with the observation of NIR phosphorescence and singlet oxygen sensitization for substituted analogues of these complexes. In contrast, dramatically lower triplet energies are predicted for ReS and ReSe corrole, as well as for ReN porphyrin, explaining their lack of phosphorescence within our spectral window, as well as their lack of singlet oxygen activity. Given that ReS and ReSe corroles exhibit relatively normal optical and electrochemical HOMO–LUMO gaps (see preceding section and Table 2), the low triplet energies can only be attributed to strong orbital relaxation effects in the triplet states. Strong evidence for such a scenario comes from scalar-relativistic B3LYP* spin density profiles of the ReS and ReSe corrole (Figure 8), which are found to be largely concentrated on the Re-chalcogenido units, even though the LUMOs of the singlet states are substantially corrole-based (Figure 7).

**Figure 8:**
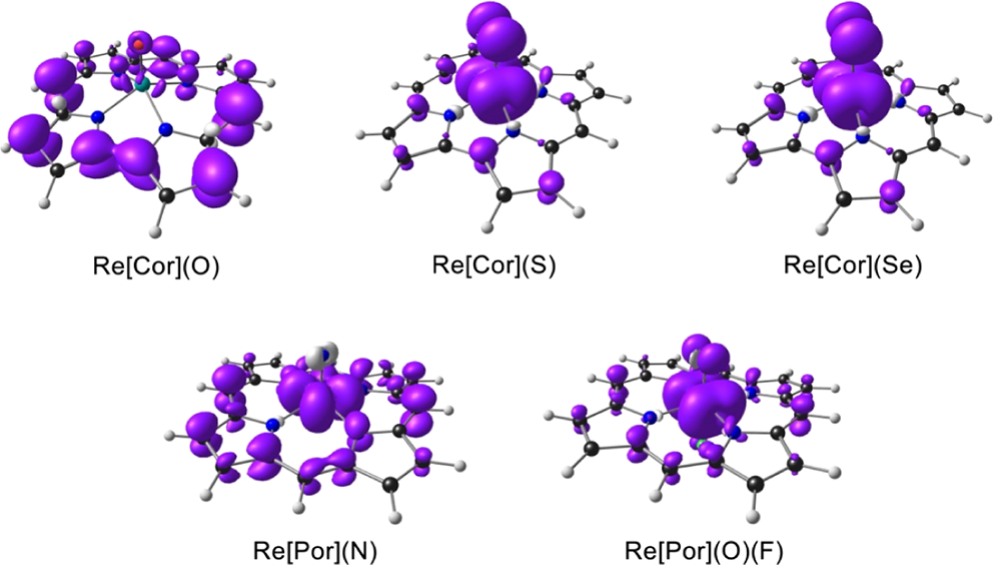
B3LYP*/STO-ZORA-TZ2P triplet spin density profiles (with a surface isovalue of 0.004 e/Å^3^) based on symmetry-unconstrained OLYP-D3 optimized geometries for *M*_*S*_ = 1.

Note that spin–orbit TDDFT calculations also yield oscillator strengths that may be expected to correlate with observed phosphorescence quantum yields (Table 3). (Since phosphorescence is an inherently spin–orbit coupling-based phenomenon, such oscillator strengths cannot be obtained scalar-relativistic TDDFT calculations.) In fact, only a qualitative correlation is observed and that too among isostructural molecules. Thus, the calculations correctly predict a higher oscillator strength for Pt­[Por] relative to Pd­[Por], consistent with observed phosphorescence quantum yields for Pt and Pd porphyrins.^56^ In the same vein, the calculations predict a higher oscillator strength for IrMe porphyrin relative to Ir­(py)_2_ corrole, again consistent with experimental results.^50–52^ For each of these pairs of complexes, the higher oscillator strength appears to be associated with a higher degree of metal character in the LUMO or in the triplet spin density, which should lead to a greater impact of spin–orbit coupling on the rate of phosphorescent emission. Certain TDDFT results, however, do not appear to correlate with experimental findings.^50–52^ Thus, unusually high phosphorescence oscillator strengths are predicted for the two Ir complexes studied, relative to most of the other metalloporphyrins and metallocorroles, which appears to be a somewhat unphysical result. It will be interesting to see whether methodological improvements yield more accurate phosphorescence oscillator strengths in the foreseeable future.

### Attempted Synthesis of Re-tellurido Corroles

Intrigued by reports of several tungsten-tellurido complexes,^70–73^ we also attempted to synthesize rhenium-tellurido corroles. Toward that end, we substituted elemental tellurium powder for selenium in the second step of the synthesis. UV–vis spectroscopy did not yield any indication of novel species; high-resolution mass spectrometry also did not indicate the formation of a ReTe corrole. Substituting TeCl_4_ for tellurium powder, and using an equimolar mixture of elemental tellurium and TeCl_4_ (which are known to react to yield TeCl_2_), proved similarly fruitless. These negative results *may* suggest that ReTe corroles are thermodynamically less stable than their lighter-chalcogen congeners (in line with tellurium’s reputation as “a maverick among the chalcogens”,^74^ a proposition that we chose to test with DFT calculations.

We accordingly revisited our earlier OLYP-D3/ZORA-STO-TZ2P calculations on the energetics of the following reactions, where {Re­[Cor]}_2_ refers to metal–metal quadruple-bonded, unsubstituted rhenium corrole dimer.

Re[Cor](O)+Me3P=1/2{Re[Cor]}2+Me3PO;ΔE=−0.59eV

Re[Cor](S)+Me3P=1/2{Re[Cor]}2+Me3PS;ΔE=−0.54eV

Re[Cor](Se)+Me3P=1/2{Re[Cor]}2+Me3PSe;ΔE=−0.48eV

Re[Cor](Te)+Me3P=1/2{Re[Cor]}2+Me3PTe;ΔE=−0.58eV

As shown above, the reaction energies (not corrected for zero-point energies) appear to be roughly independent of the chalcogen. The result, however, does not imply that the Re-chalcogenido corroles are all equally stable, but rather that their stabilities track those of the corresponding phosphine chalcogenides. The latter is a most valuable clue: while phosphine oxides, sulfides, and selenides are stable compounds,^75,76^ phosphine tellurides are known to be thermodynamically unstable (even though a handful have been reported).^77–80^ At this point, our failure to generate ReTe corroles may reflect their limited stability. That said, we have far from exhausted all reasonable avenues that could lead to the successful isolation of ReTe corroles.

## Concluding Remarks

The main conclusions of this study are as follows.

1. The high-temperature insertion of rhenium into free-base *meso*-triarylcorroles, followed by exposure to elemental selenium, has yielded a series of stable rhenium-selenido corroles in 70–75% yields. The synthesis is similar to that reported recently for rhenium-sulfido corroles. The stability of ReO, ReS, and ReSe corroles is a testament to rhenium’s chalcophilic character, specifically its unique status as a metal that is almost equally oxophilic and thiophilic. Rhenium-tellurido corroles, however, resisted synthesis, a reflection, potentialy, of their limited stability.

2. Unlike ReO corroles, which exhibit room-temperature phosphorescence and efficiently sensitize singlet oxygen formation, ReS and ReSe corroles proved nonemissive in the NIR-I regime and also did not exhibit singlet oxygen formation; a similar negative result was also obtained for ReN porphyrins. Whether some of these complexes might emit in the NIR-II regime remains an interesting question for the future.

3. Based on a relatively wide-ranging DFT and TDDFT survey of 4d and 5d metalloporphyrins and metallocorroles, the lack of near-infrared (<1000 nm) phosphorescence and singlet oxygen activity of ReS and ReSe corroles, and of ReN porphyrins, may be attributed to low triplet-state energies that are insufficient for singlet oxygen generation. Such calculations are likely to aid in the design of new triplet emitters and photosensitizers.

## Experimental Section

### Materials and Methods

The majority of chemicals were purchased from Merck. Free-base triarylcorroles were prepared according to literature procedures.^81,82^ Rhenium-nitride porphyrins studied were prepared according to literature methods in two steps. The first step involved refluxing trichlorobenzene solutions of free-base porphyrins and ReCl_5_ to give rhenium-oxido porphyrins.^83^ The second step involved heating chloroform-ethanol solutions of rhenium-oxido porphyrins and hydrazine hydrate to yield rhenium-nitrido porphyrins.^33^

UV–visible-NIR spectra were recorded on a Cary 8454 spectrophotometer. ^1^H NMR spectra were recorded on a 400 MHz Bruker Avance III HD spectrometer equipped with a 5 mm BB/1H SmartProbe at 298 K in CDCl_3_ and 243 K in CD_2_Cl_2_ and referenced to residual CHCl_3_ at 7.26 ppm and CH_2_Cl_2_ at 5.31 ppm. High-resolution electrospray ionization mass spectra (HR-ESI-MS) were recorded on an LTQ Orbitrap XL spectrometer in the positive mode.

Cyclic voltammetry was carried out at ambient temperature with a Gamry Reference 620 potentiostat equipped with a three-electrode system: a 3 mm disk glassy carbon working electrode, a platinum wire counter electrode, and a saturated calomel reference electrode (SCE).Tetra­(*n*-butyl)­ammonium perchlorate was used as the supporting electrolyte. Anhydrous CH_2_Cl _2_ (Aldrich) was used as the solvent. The electrolyte solution was purged with argon for at least 2 min prior to all measurements, which were carried out under an argon blanket. The glassy carbon working electrode was polished using a polishing pad and 0.05-μm polishing alumina from ALS, Japan. All potentials were referenced to the SCE.

#### Re­[T*p*XPC]­(Se)

To a 50 mL two-necked round-bottom flask fitted with a reflux condenser and containing decalin (15 mL) and a magnetic stirring bar were added a free-base corrole, H_3_[T*p*XPC] (0.17 mmol), Re_2_(CO)_10_ (221.8 mg, 0.34 mmol), and potassium carbonate (140 mg). The contents were deoxygenated with a flow of argon and then refluxed overnight with constant stirring under argon. Phosphorus trichloride (148.7 μL, 10 equiv) and elemental selenium, powder (13.4 mg, 5 equiv) were then added and the reaction was continued under reflux (i.e., at ∼ 180 °C) for ∼ 4 h. The color of the reaction slowly turned to brown and completion of the reaction was monitored by UV–vis spectroscopy and mass spectrometry. Upon cooling, the reaction mixture was loaded directly on to a silica gel column with *n*-heptane as the mobile phase. The decalin was first removed by eluting with pure *n*-heptane. Different solvent mixtures were then used to elute the various Re­[T*p*XPC]­(Se) derivatives: 3:1 *n*-heptane/dichloromethane for X = CF_3_, H, CH_3_ and F and 1:1 *n*-heptane/dichloromethane for X = OCH_3_. All fractions with λ_max_ ∼ 393 nm were collected and evaporated to dryness. The products were further purified with a second round of column chromatography and finally with preparative thin-layer chromatography, all with the same solvent system as in the first round. Yields and analytical details for the different complexes are given below. Note that the NMR assignments (*o*1, *o*2) and (*m*1, *m*2) refer to symmetry-distinct (diastereotopic) *ortho* and *meta* protons for a given aryl group.

#### Re­[T*p*CF_3_PC]­(Se)

Yield 123.2 mg (0.124 mmol, 72.9%). UV–vis (CH_2_Cl_2_) λ_max_ [nm, ε x 10^–4^ (M^–1^cm^–1^)]: 280 (5.25), 344 (3.20), 393 (4.82), 471 (2.77), 577 (1.68). ^1^H NMR (400 MHz, CD_2_Cl_2_, – 30 °C): δ 9.64 (d, 2H, ^3^ *J*_HH_ = 4.4 Hz, β-H); 9.31 (d, 2H, ^3^ *J*_HH_ = 4.6 Hz, β-H); 9.27 (d, 2H, ^3^ *J*_HH_ = 4.9 Hz, β-H); 9.11 (d, 2H, ^3^ *J*_HH_ = 4.6 Hz, *β-H*); 8.74 (d, 2H, ^3^ *J*_HH_ = 7.8 Hz, 5,15-*o*1-Ph); 8.64 (d, 1H, ^3^ *J*_HH_ = 7.8 Hz. 10-*o*1-Ph); 8.19 (d, 2H, ^3^ *J*_HH_ = 8.1 Hz 5,15-*m*1-Ph); 8.13 (t, 3H, ^3^ *J*_HH_ = 8.7 Hz, 5,15*-o*2*-*Ph 10-*m*1-Ph); 8.03 (t, 2H, ^3^ *J*_HH_ = 8.1 Hz 5,15*-*Ph); 7.97 (s, 2H, 10-*m*2 and *o*2-Ph). MS (ESI): [M+H]^+^ = 995.0338 (expt), 995.0342 (calcd for M = C_40_H_20_N_4_F_9_SeRe).

#### Re­[T*p*FPC]­(Se)

Yield 108.3 mg (0.128 mmol, 75.4%). UV–vis (CH_2_Cl_2_): λ_max_ (nm), [ε x 10^–4^ (M^–1^cm^–1^)]: 280 (5.04), 344 (3.18), 391 (4.76), 471 (2.60); 574(1.59). ^1^H NMR (400 MHz, CD_2_Cl_2_, – 30 °C): δ 9.63 (d, 2H, ^3^ *J*_HH_ = 4.5 Hz, β-H); 9.31 (d, 2H, ^3^ *J*_HH_ = 4.5 Hz, β-H); 9.28 (d, 2H, ^3^ *J*_HH_ = 5.0 Hz, β-H); 9.11 (d, 2H, ^3^ *J*_HH_ = 5.0 Hz, β-H); 8.58 (t, 2H, ^*3*^ *J*_HH_ = 7.1 Hz, 5,15-*o*1-Ph); 8.49 (d, 1H, ^*3*^ *J*_HH_ = 7.1 Hz, 10-*o*1-Ph); 7.97 (t, 2H, ^*3*^ *J*_HH_ = 7.4 Hz, 5,15-*o*2-Ph); 7.80 (t, 2H, ^3^ *J*_HH_ = 7.2 Hz, 10-o2-Ph); 7.67 – 7.55 (m, 3H, 5,10,15-*m*1-Ph); 7.48 (td, 2H, ^*3*^ *J*_HH_ = 8.8, 2.6 Hz, 10-*m*2-Ph); 7.41 (dt, 1H, ^3^ *J*_HH_= 8.7, 4.9 Hz, 10-*m*2-Ph). MS (ESI): [M+H]^+^ = 845.043 (expt), 845.0437 (calcd for M = C_37_H_20_F_3_N_4_SeRe).

#### Re­(TPC)­(Se)

Yield 96.4 mg (0.122 mmol, 71.7%). UV–vis (CH_2_Cl_2_): λ_max_ (nm), [ε x 10^–4^ (M^–1^cm^–1^)]: 282 (4.74), 345 (3.17), 393 (4.40), 430 (3.03); 577 (1.59). ^1^H NMR (400 MHz, CD_2_Cl_2_, – 30 °C): δ 9.67 (d, 2H, ^3^ *J*_HH_ = 4.5 Hz, β-H); 9.36 (d, 2H, ^3^ *J*_HH_ = 4.6 Hz, β-H); 9.31 (d, 2H, ^3^ *J*_HH_ = 4.9 Hz, β-H); 9.13 (d, 2H, ^3^ *J*_HH_ = 4.9 Hz, β-H); 8.63 (d, 2H, *J* = 7.6 Hz, 5,15-*o*1-Ph); 8.53 (d, 1H, *J* = 7.5 Hz, 10-*o*1-Ph); 8.04 (d, 2H, *J* = 6.9 Hz, 5,15-*o*2-Ph); 7.94 (t, 2H, *J* = 7.3 Hz, 5,15-m2-Ph); 7.90 – 7.77 (m, 8H, 10-o2-Ph, 5,10,15-*p*-Ph and 5,15-m2-Ph); 7.73 (d, 1H, *J* = 7.5 Hz, 10-*m*2-Ph). MS (ESI): [M+H]^+^ = 791.0718 (expt), 791.0720 (calcd for M = C_37_H_23_N_4_SeRe).

#### Re­[T*p*CH_3_PC]­(Se)

Yield 104.0 mg (0.125 mmol, 73.5%). UV–vis (CH_2_Cl_2_): λ_max_ (nm), [ε x 10^–4^ (M^–1^cm^–1^)]: 280 (4.74), 343 (2.97), 339 (2.86), 393 (4.41), 438 (3.29); 577 (1.51). ^1^H NMR (400 MHz, CD_2_Cl_2_, – 30 °C): δ 9.64 (d, 2H, ^3^ *J*_HH_ = 4.5 Hz, β-H); 9.34 (d, 2H, ^3^ *J*_HH_ = 4.4 Hz, β-H); 9.30 (d, 2H, ^3^ *J*_HH_ = 4.9 Hz, β-H); 9.13 (d, 2H, ^3^ *J*_HH_ = 4.9 Hz, β-H); 8.50 (dd, 2H, ^3^ *J*_HH_ = 7.7, 2.0 Hz, 5,15-*o*1-Ph); 8.39 (dd, 1H, ^3^ *J*_HH_ = 7.6, 2.0 Hz, 10-*o*1-Ph); 7.92 (dd, 2H, ^3^ *J*_HH_ = 7.6, 1.9 Hz, 5,15-*o*2-Ph); 7.74 (d, 3H, ^3^ *J*_HH_ = 7.4 Hz, 5,15-m1-Ph); 7.71 (d, 1H, ^3^ *J*_HH_ = 6.36 Hz, 10-*o*2-Ph); 7.68 (d, 2H, ^3^ *J*_HH_ = 7.8 Hz, 10-*m*2-Ph); 7.58 (d, 2H, ^3^ *J*_HH_ = 7.8 Hz, 5,15-*m*2-Ph); 7.51 (d, 1H, ^3^ *J*_HH_ = 7.8 Hz, 10-*m*2-Ph); 2.68 (s, 6H, 5,15-*p*-CH_3_); 2.66 (s, 3H, 10-*p*-CH_3_). MS (ESI): [M+H]^+^ = 833.1185 (expt), 833.1190 (calcd for M = C_40_H_29_N_4_SeRe).

#### Re­[T*p*OCH_3_PC]­(Se)

Yield 104.7 mg (0.119 mmol, 70.0%). UV–vis (CH_2_Cl_2_): λ_max_ (nm), [ε x 10^–4^ (M^–1^cm^–1^)]: 283 (5.13), 346 (3.21), 393 (4.66), 439 (3.41); 578 (1.55). ^1^H NMR (400 MHz, – 30 °C): δ 9.63 (d, 2H, ^3^ *J*_HH_ = 4.5 Hz, β-H); 9.34 (d, 2H, ^3^ *J*_HH_ = 4.4 Hz, β-H); 9.32 (d, 2H, ^3^ *J*_HH_ = 5.0 Hz, β-H); 9.15 (d, 2H, ^3^ *J*_HH_ = 4.9 Hz, β-H); 8.54 (dd, 2H, ^3^ *J*_HH_ = 8.4, 2.3 Hz, 5,15-*o*1-Ph); 8.42 (dd, 1H, ^3^ *J*_HH_ = 8.3, 2.3 Hz, 10-*o*1-Ph); 7.96 (dd, 2H, ^3^ *J*_HH_ = 8.4, 2.3 Hz, 5,15-*o*2-Ph); 7.77 (dd, 1H, ^3^ *J*_HH_ = 8.3, 2.3 Hz, 10-*o*2-Ph); 7.45 (dd, 2H, ^3^ *J*_HH_ = 8.4, 2.8 Hz, 5,15-*m*1-Ph); 7.39 (dd, 1H, ^3^ *J*_HH_ = 8.4, 2.7 Hz, 10-*m*1-Ph); 7.30 (dd, 2H, ^3^ *J*_HH_ = 8.4, 2.7 Hz, 5,15-*m*2-Ph); 7.23 (d, 1H, ^3^ *J*_HH_ = 8.4, 2.7 Hz, 10-*m*2-Ph); 4.06 (s, 6H, 5,15*-p*-OCH_3_); 4.04 (s, 3H, 10-*p*-OCH_3_). MS (ESI): [M+H]^+^ = 881.1035 (expt), 881.1038 (calcd for M = C_40_H_29_O_3_N_4_SeRe).

### Sample Preparation for Crystallography

Crystals were grown by slow diffusion of methanol into concentrated solutions of the samples in dichloromethane.

### Single-Crystal X-ray Diffraction Analyses

X-ray data were collected on beamline 12.2.1 at the Advanced Light Source, Lawrence Berkeley National Laboratory. Samples were mounted on MiTeGen Kapton loops and placed in a 100(2) K nitrogen cold stream provided by an Oxford Cryostream 800 Plus low-temperature apparatus on the goniometer head of a Bruker D8 diffractometer equipped with a PHOTON II CPAD detector operating in shutterless mode. Diffraction data were collected using synchrotron radiation monochromated using silicon(111) to a wavelength of 0.7288(1)­Å. An approximate full-sphere of data was collected using a combination of φ and ω scans with scan speeds of one second per degree. The structures were solved by intrinsic phasing (SHELXT^84^ and refined by full-matrix least-squares on F2 (SHELXL-2014.^85^ All non-hydrogen atoms were refined anisotropically. Hydrogen atoms were geometrically calculated and refined as riding atoms.

### Photophysical Studies

The complexes in question were screened for potential emission using a Fluorolog 3 spectrometer, with a setup identical to that reported previously.^62^ Singlet oxygen assays were also performed as described in the same report.

### Computational Methods

All geometry optimizations were carried out with the scalar-relativistic zeroth-order regular approximation (ZORA) Hamiltonian,^86^ OLYP^87,88^ functional, Grimme’s D3 ^89^ dispersion correction, ZORA TZ2P all-electron relativistic basis sets, carefully tested fine integration grids and tight criteria for the SCF cycles and geometry optimizations, as implemented in the ADF 2019 program system.^90^ Time-dependent B3LYP*^68^ calculations were carried out on OLYP optimized geometries using both scalar-relativistic (SR) and spin–orbit (SO) ZORA Hamiltonians.

## Supplementary Material



## Data Availability

All data generated or analyzed in this study are included in this published article and its Supporting Information.

## Notes

Mass spectra, selected photophysical data, and optimized DFT coordinates (PDF)
