# Supplementary material for: Rhenium-Selenido Corroles: Reflections on 5d Metalloporphyrins and Metallocorroles as Triplet Emitters and Photosensitizers
Source: Inorg Chem. 2025 Jun 9;64(24):12242–53. doi: 10.1021/acs.inorgchem.5c01593 (PMC12188564; doi:10.1021/acs.inorgchem.5c01593)
Supplement: Supplementary file 1 [file ic5c01593_si_001.pdf]

Supporting Information

Rhenium-Selenido Corroles: Reflections on 5d Metalloporphyrins and Metallocorroles as Triplet Emitters and Photosensitizers

Abraham B. Alemayehu,[a] Jeanet Conradie,[a,b] Simon Larsen [a] Bjørn Cicerôn Lukas Pérez,[a] Nicholas S. Settineri,[c] and Abhik Ghosh*,[a]

[a] Department of Chemistry, University of Tromsø, N-9037 Tromsø, Norway;

email: abhik.ghosh@uit.no (AG);

[b] Department of Chemistry, University of the Free State, P.O. Box 339, Bloemfontein 9300, Republic of South Africa;

[c] Advanced Light Source, Lawrence Berkeley National Laboratory, Berkeley, CA 94720-8229, United States.

Table of Contents

A. ^{1}H NMR spectra 3
B. Electrospray ionization mass spectra 8
C. DFT OLYP-D3/STO-ZORA-TZ2P MO energy level diagrams 11
D. DFT OLYP-D3/STO-ZORA-TZ2P frontier MOs 13
F. Optimized OLYP-D3/STO-ZORA-TZ2P Cartesian coordinates (Å) 17
Re porphyrins and corroles, $M_S = 0$ 17
1. Re[Cor](O), C_s 17
2. Re[Cor](S), C_s 18
3. Re[Cor](Se). C_s 18
4. Re[Por](N), C_{4v} 19
5. Re[Por](O)(F), C_{4v} 20
Other metallocorroles, $M_S = 0$ 21
6. Ru[Cor](N). C_s 21
7. Os[Cor](N), Cs 22
8. Ir[Cor](py)$_2$, C_s 22
9. Pt[Cor](Ph)(py), C_s 24
10. Au[Cor], C_{2v} 25
Other metalloporphyrins, $M_S = 0$ 26
11. Ir[Por](Me), C_s 26
12. Pd[Por], D_{4h} 27
13. Pt[Por], D_{4h} 27

Re porphyrins and corroles, $M_S = 1$ 28
14. Re[Cor](O), C_1 28
15. Re[Cor](S), C_1 29
16. Re[Cor](Se), C_1 30
17. Re[Por](N), C_1 31
18. Re[Por](O)(F), C_1 31
Other metallocorroles, $M_S = 1$ *32*
19. Ru[Cor](N), C_1 32
20. Os[Cor](N), C_1 33
21. Ir[Cor](py)$_2$, C_1 34
22. Pt[Cor](Ph)(py), C_1 35
23. Au[Cor], C_1 36
Other metalloporphyrins, $M_S = 1$ 37
24. Ir[Por](Me), C_1 37
25. Pd[Por], C_1 38
26. Pt[Por], C_1 39

A. ^{1}H NMR spectra

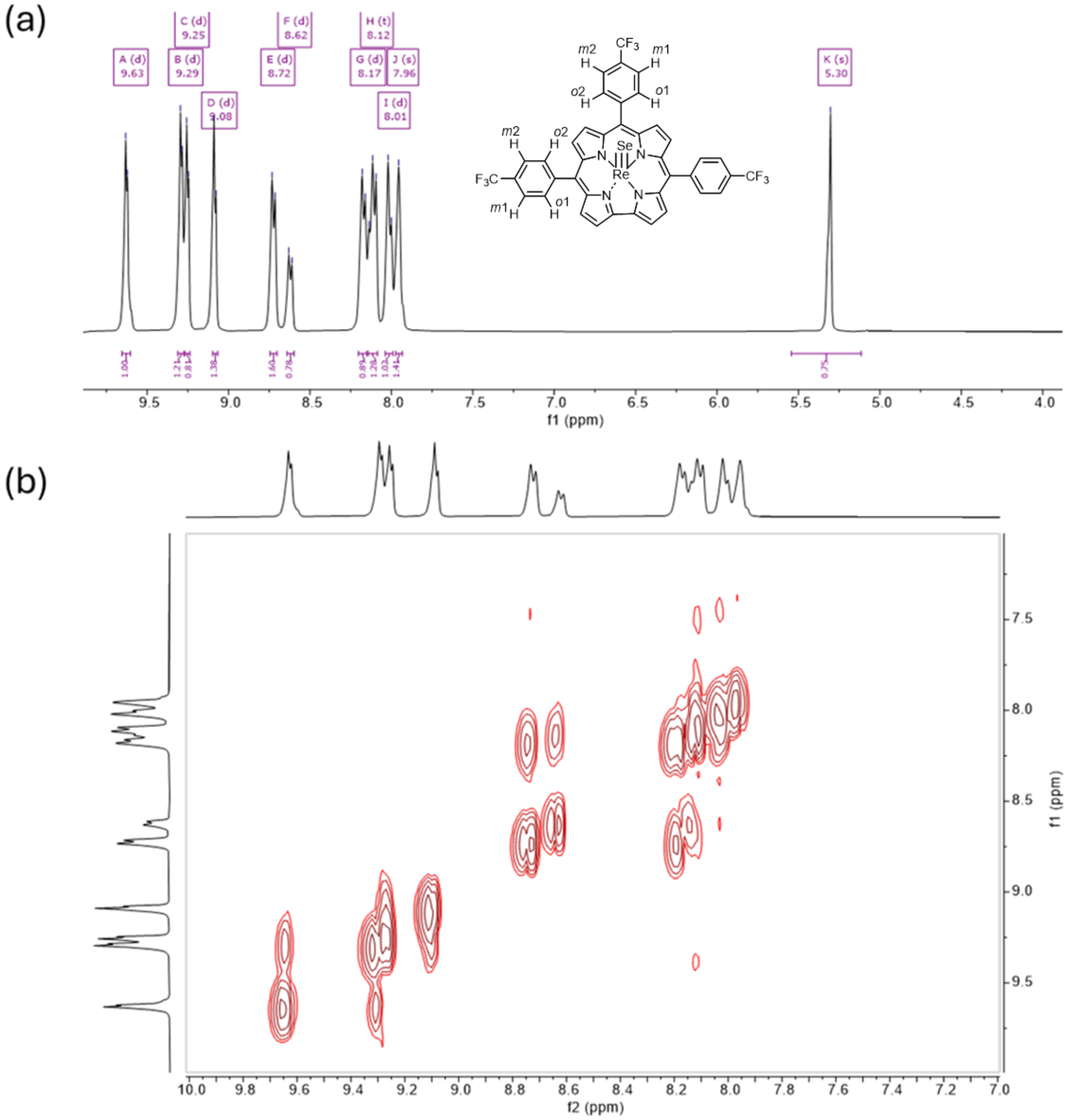

Figure S1. ^{1}H NMR spectra (400 MHz, CD_2Cl_2, 243 K) of Re[T*p*CF_3PC](Se): (a) 1D spectrum and (b) ^{1}H-^{1}H COSY.

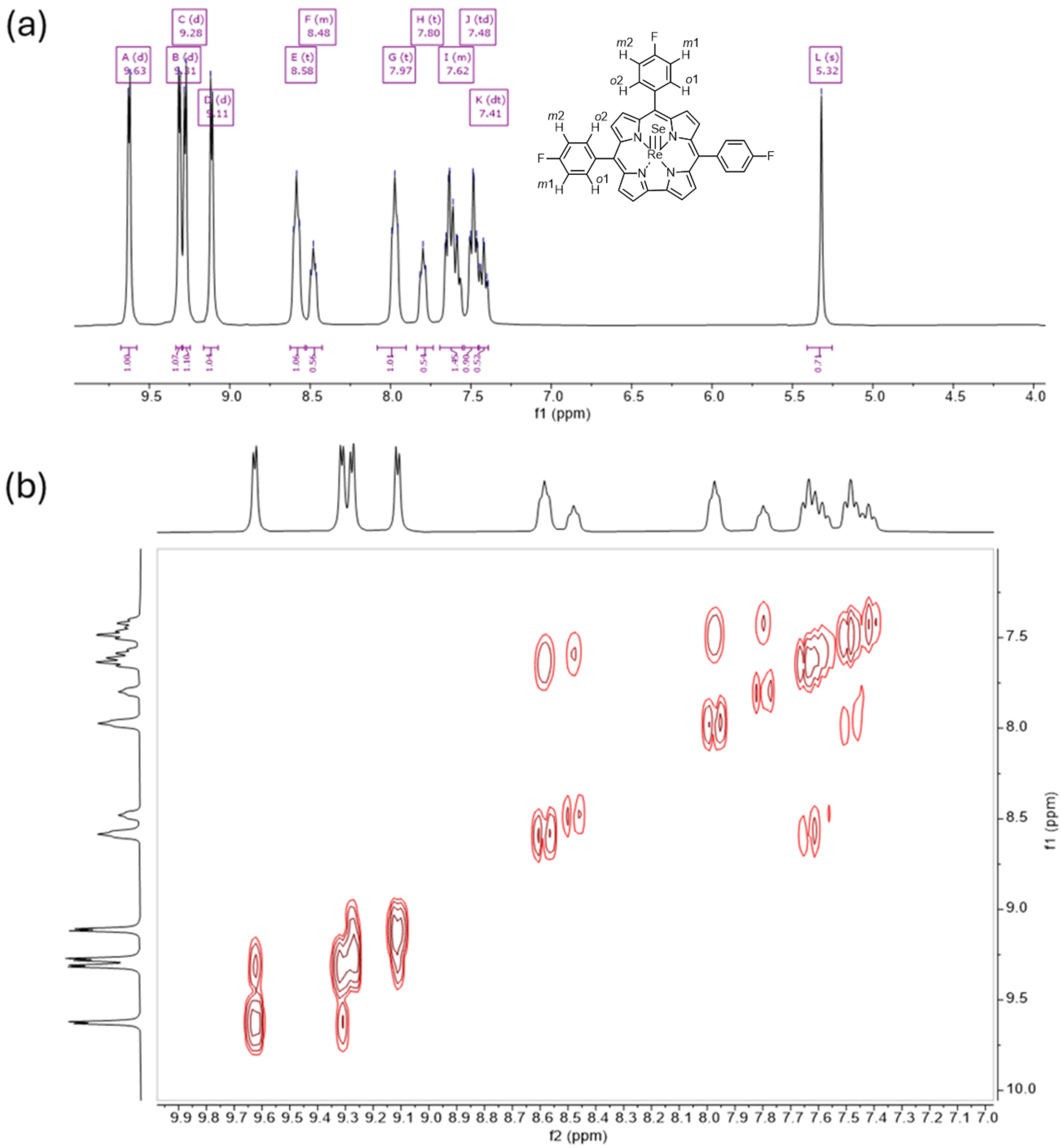

Figure S2. ^{1}H NMR spectra (400 MHz, CD_2Cl_2, 243 K) of Re[T*p*FPC](Se): (a) 1D spectrum and (b) ^{1}H-^{1}H COSY.

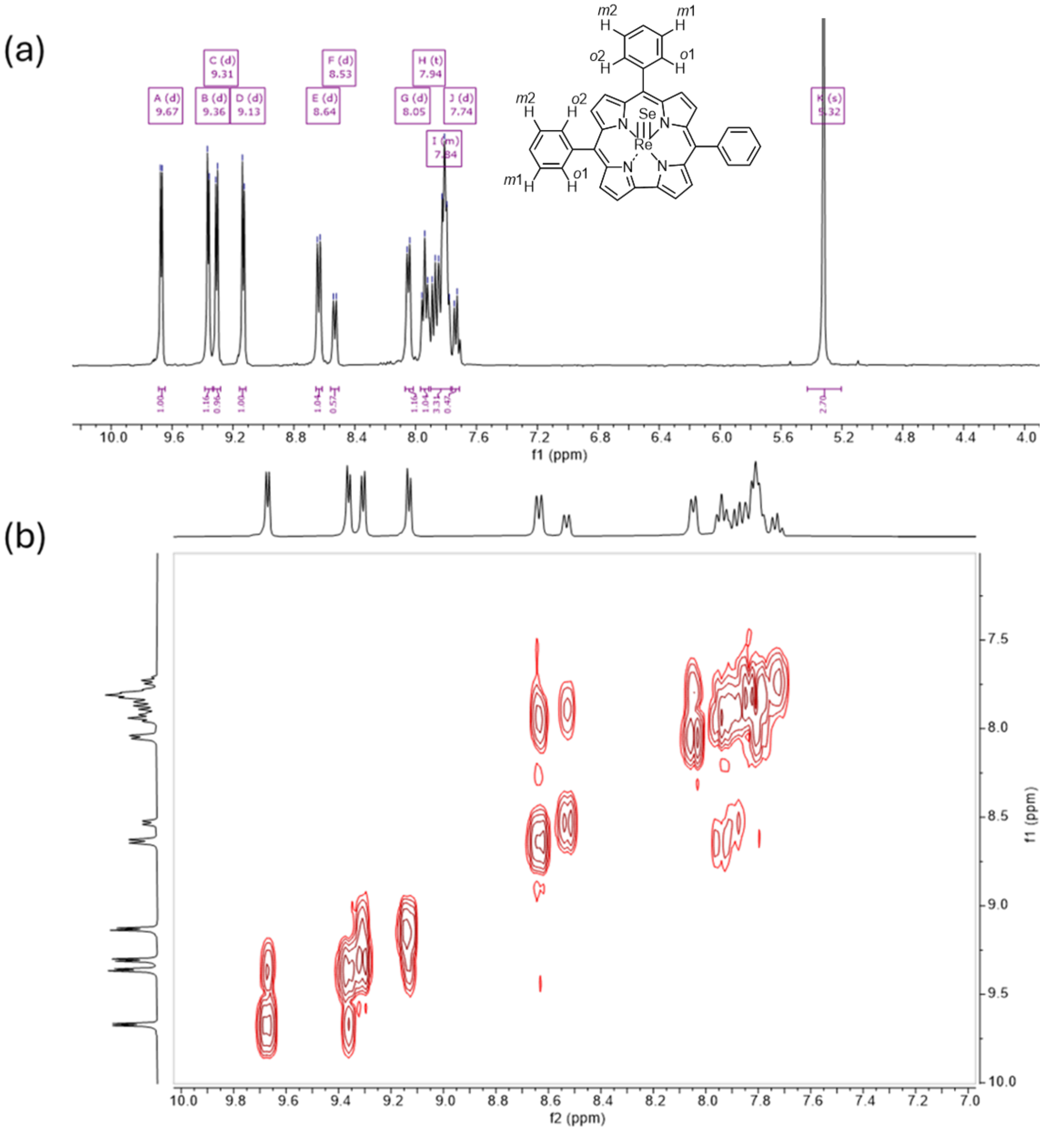

Figure S3. ^{1}H NMR spectra (400 MHz, CD_2Cl_2, 243 K) of Re[TPC](Se): (a) 1D spectrum and (b) ^{1}H-^{1}H COSY.

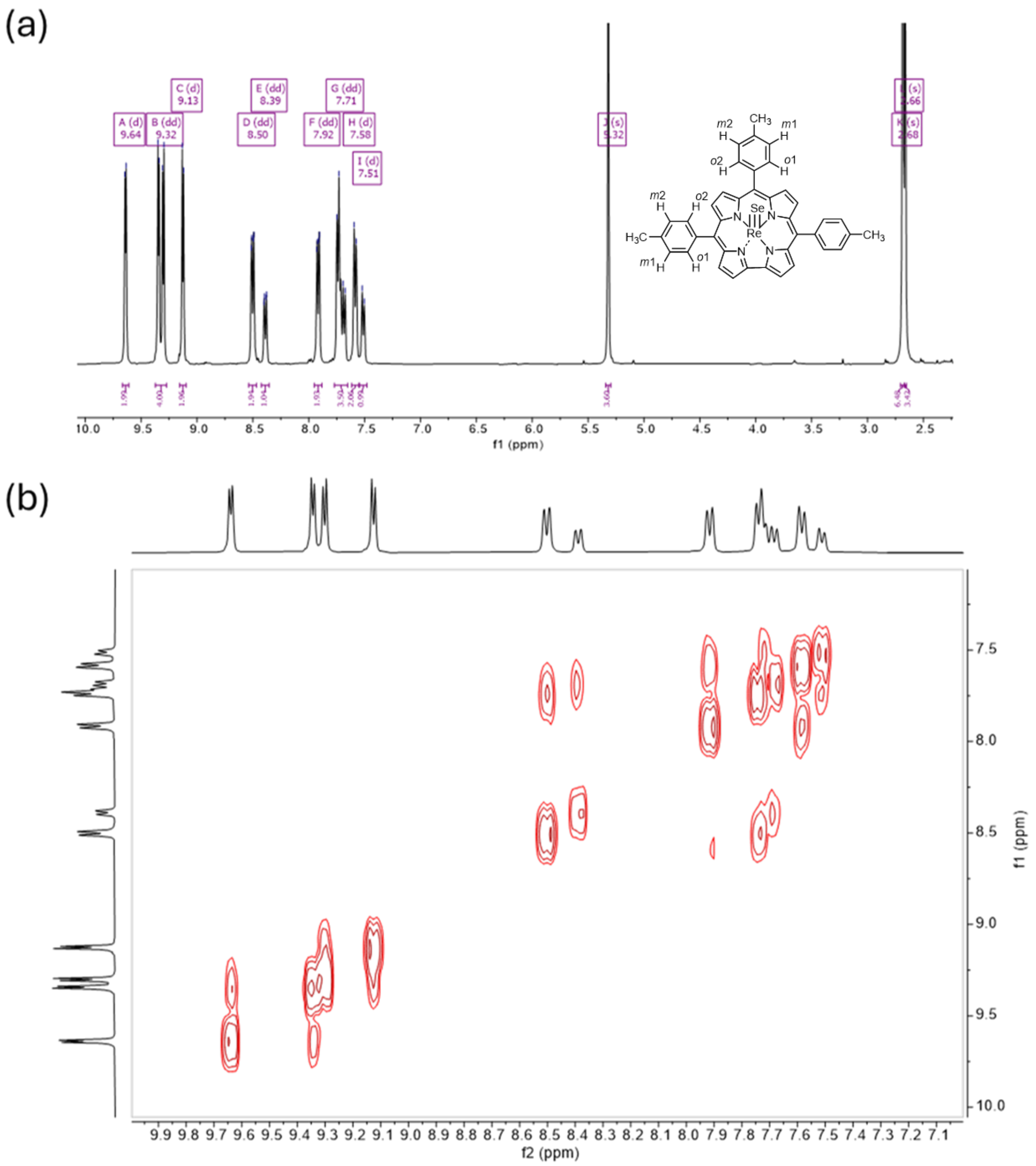

Figure S4. ^{1}H NMR spectra (400 MHz, CD_2Cl_2, 243 K) of Re[T*p*CH_3PC](Se): (a) 1D spectrum and (b) ^{1}H-^{1}H COSY.

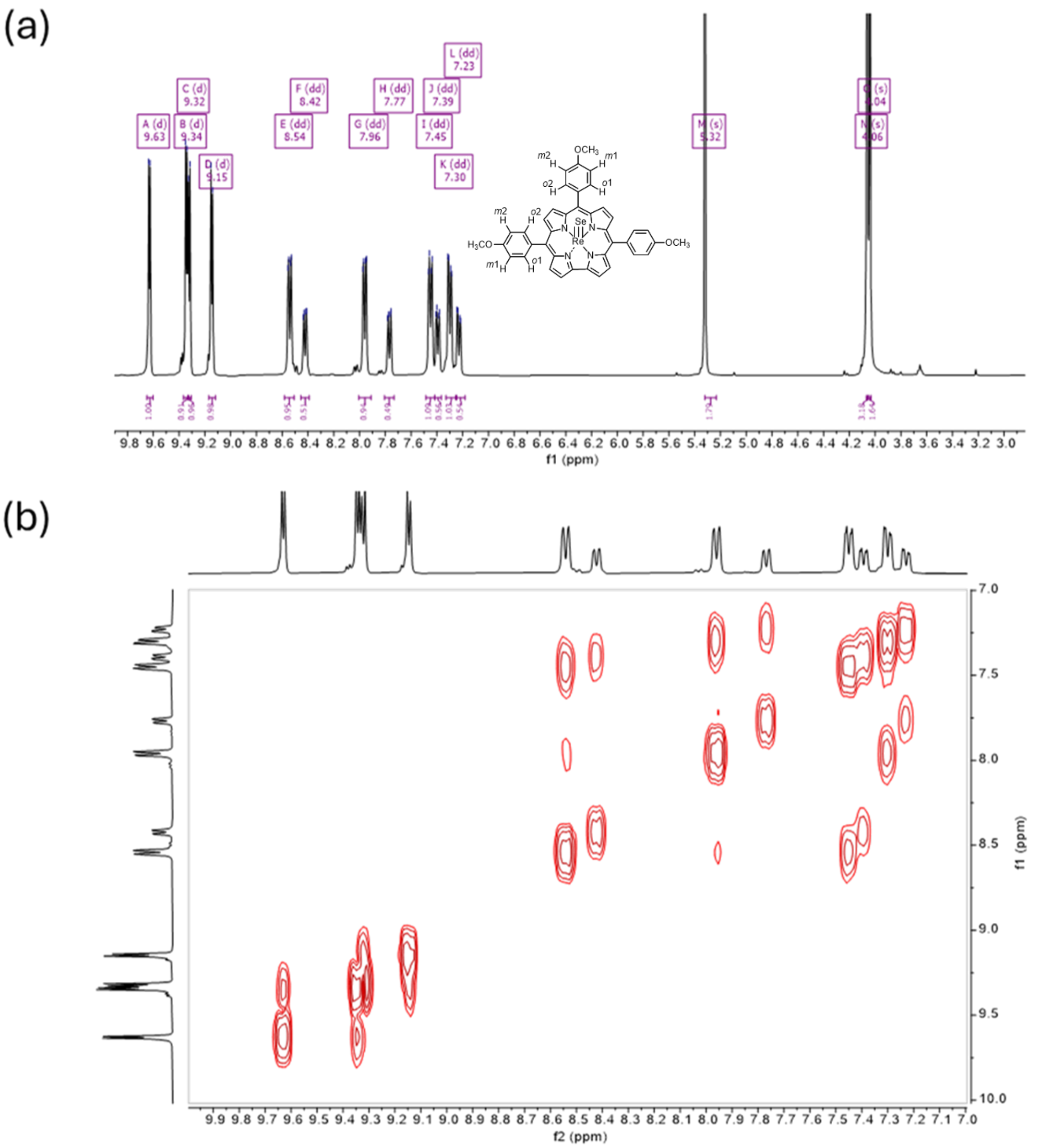

Figure S5. ^{1}H NMR spectra (400 MHz, CD_2Cl_2, 243 K) of Re[T*p*OCH_3PC](Se): (a) 1D spectrum and (b) ^{1}H-^{1}H COSY.

B. Electrospray ionization mass spectra

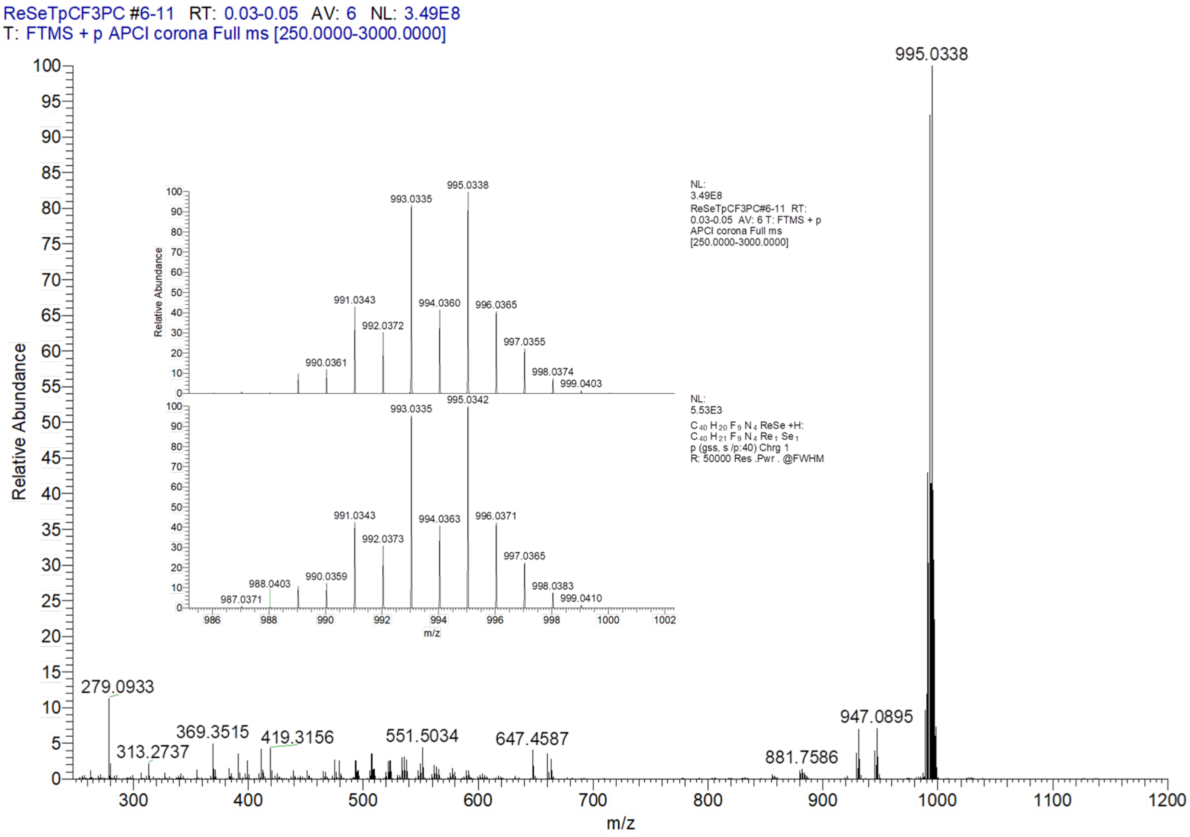

Figure S6. HR-ESI-MS of Re[T*p*CF$_3$PC](Se). Inset: experimental molecular ion peak (above) and simulation (below).

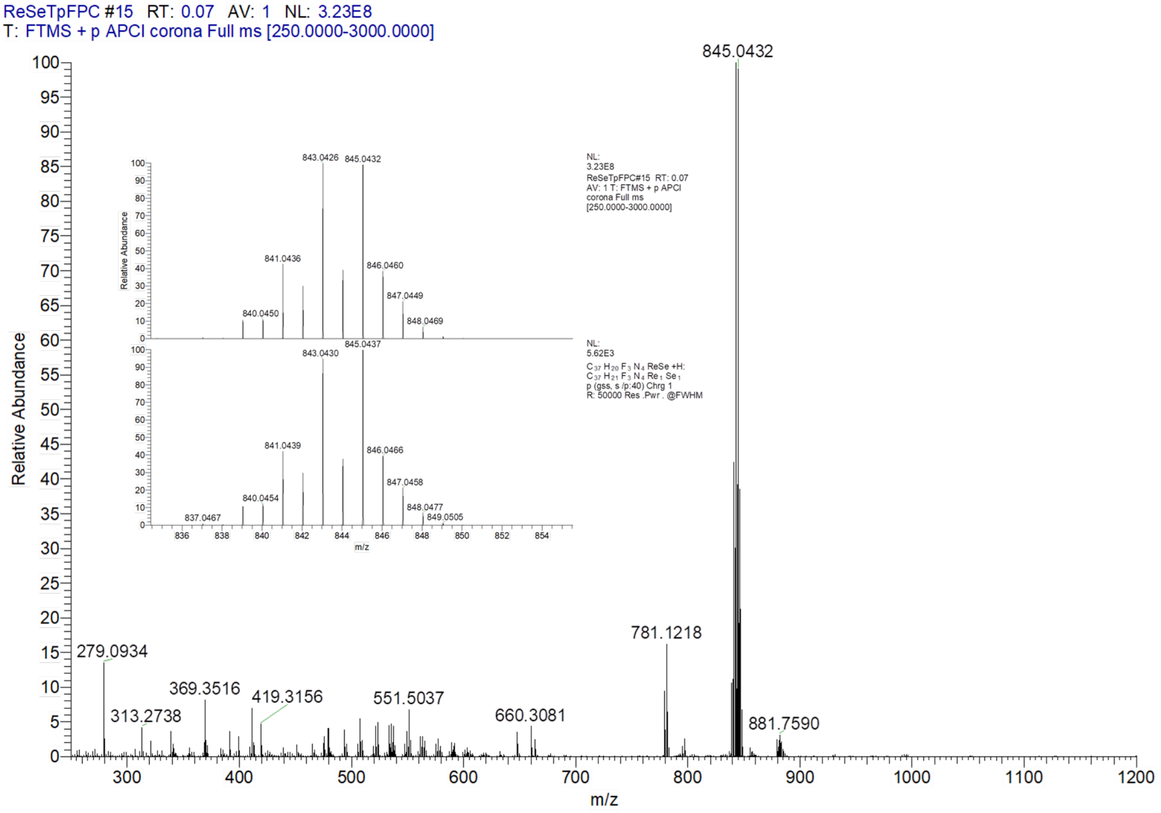

Figure S7. HR-ESI-MS of Re[T*p*FPC](Se). Inset: experimental molecular ion peak (above) and simulation (below).

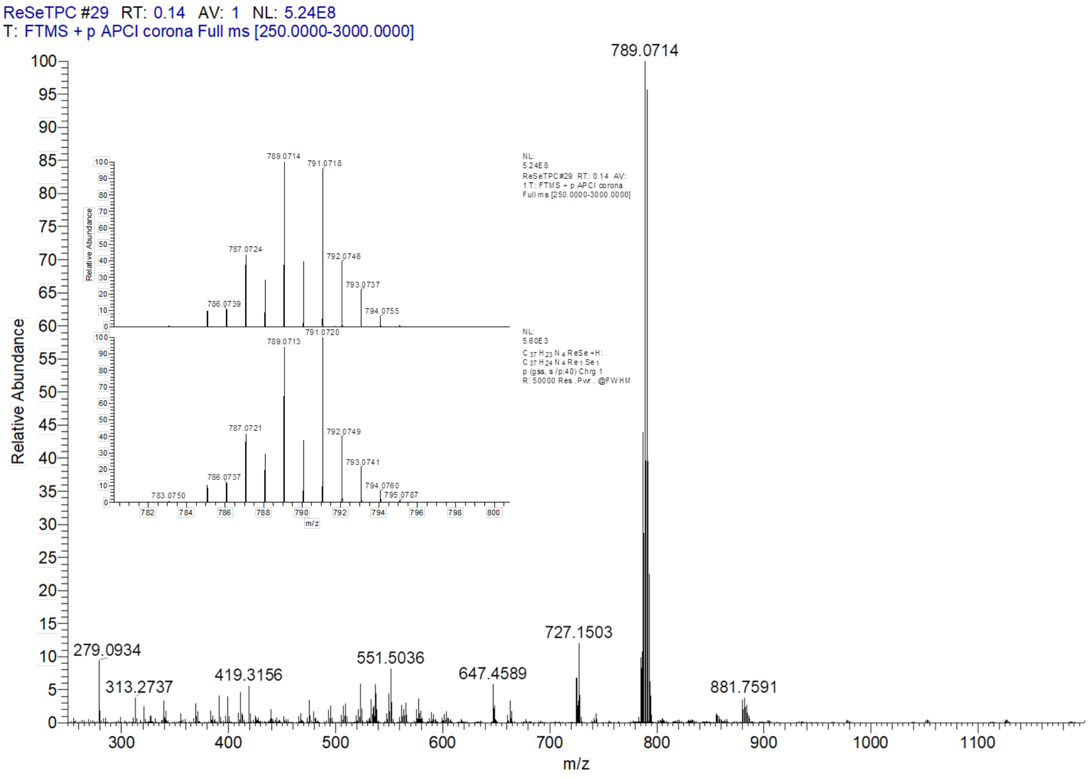

Figure S8. HR-ESI-MS of Re[TPC](Se). Inset: experimental molecular ion peak (above) and simulation (below).

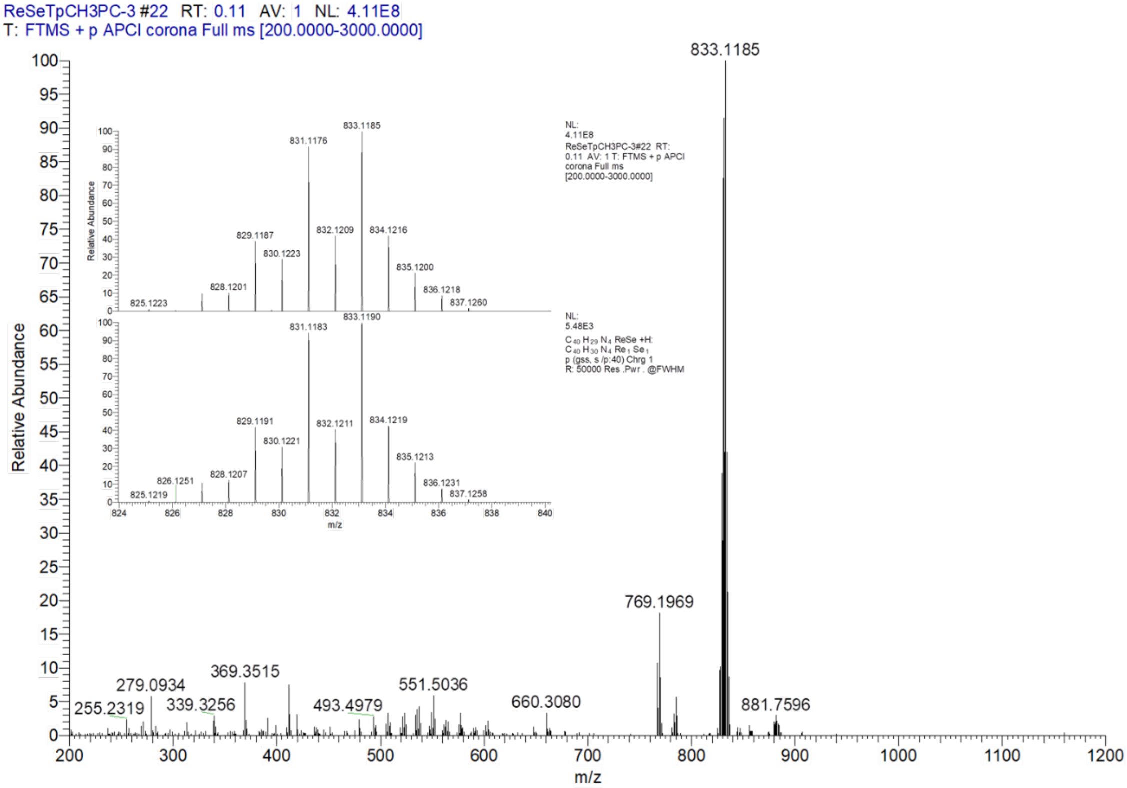

Figure S9. HR-ESI-MS of Re[T*p*CH$_3$PC](Se). Inset: experimental molecular ion peak (above) and simulation (below).

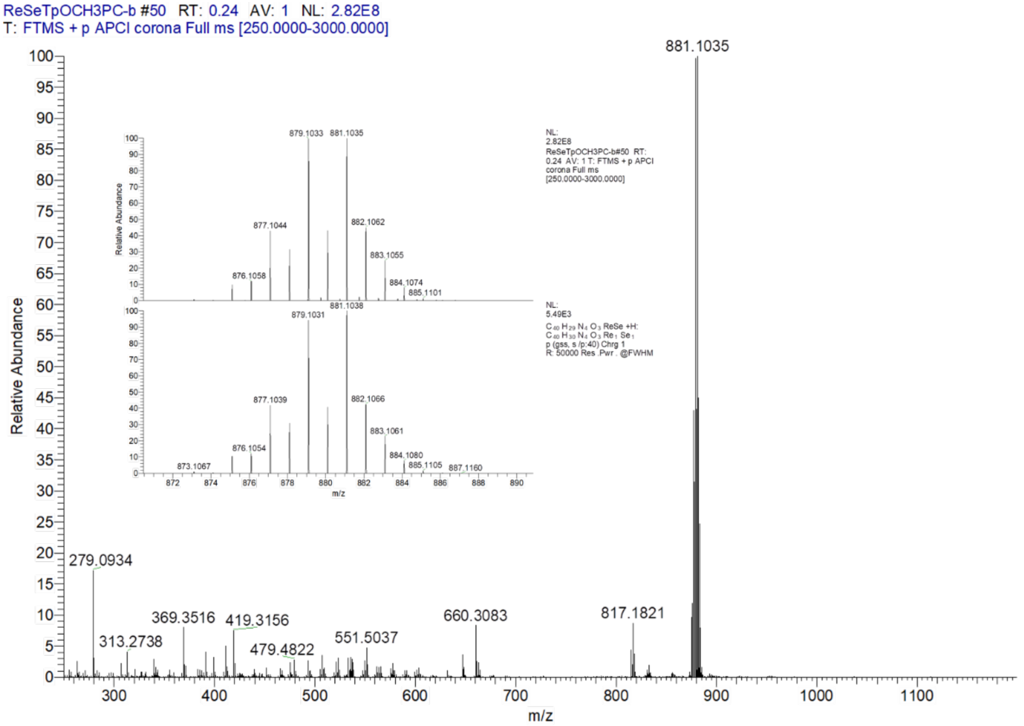

Figure S10. HR-ESI-MS of Re[T*p*OCH$_3$PC](Se). Inset: experimental molecular ion peak (above) and simulation (below).

C. DFT OLYP-D3/STO-ZORA-TZ2P MO energy level diagrams

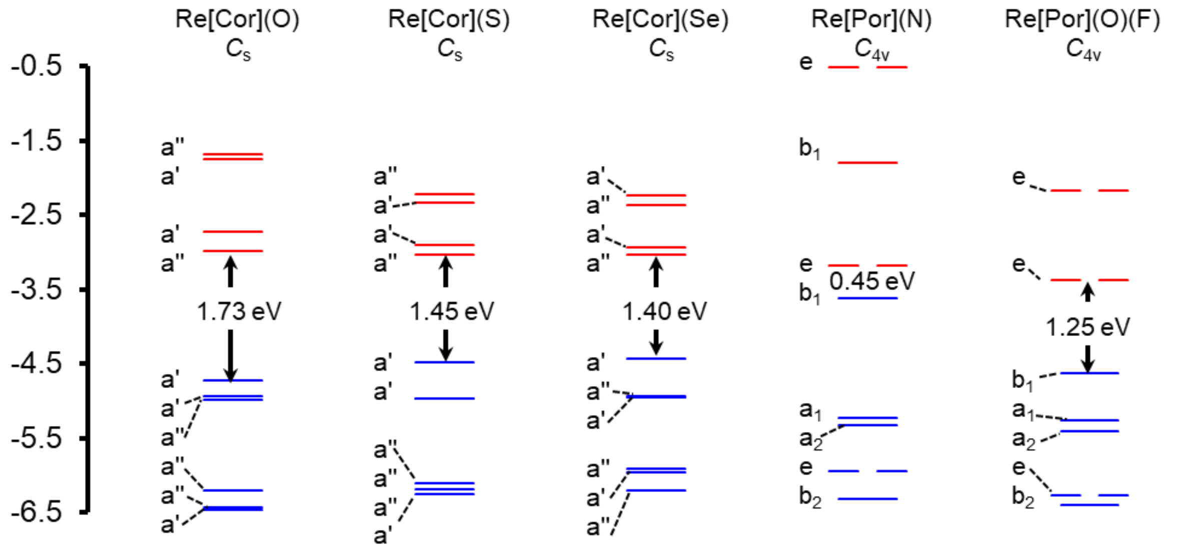

Figure S11. OLYP-D3/STO-ZORA-TZ2P MO energy level diagrams of Re porphyrins and corroles based on ground-state optimized geometries.

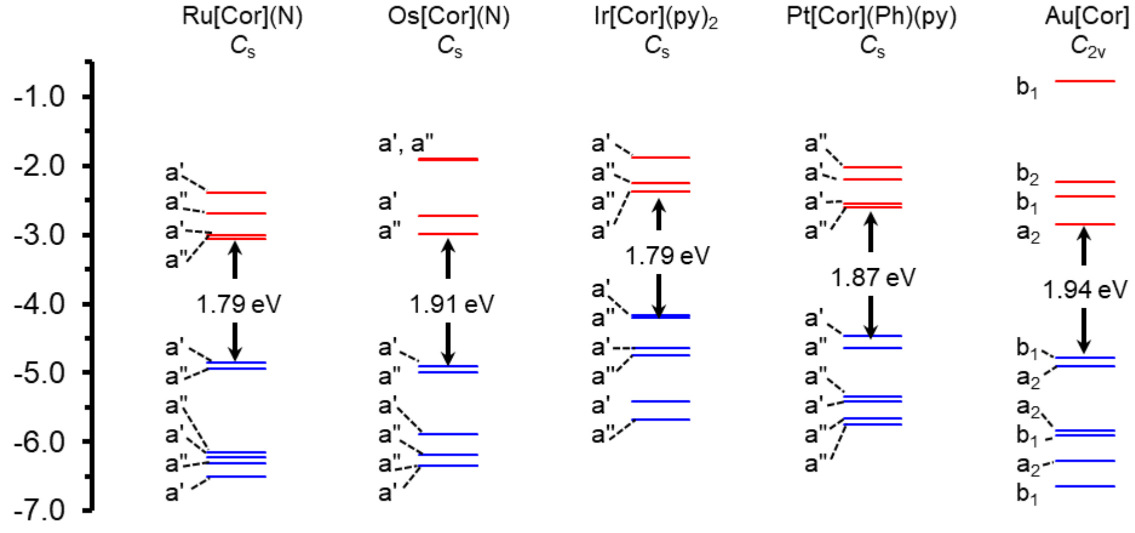

Figure S12. OLYP-D3/STO-ZORA-TZ2P MO energy level diagrams of metallocorroles based on ground-state optimized geometries.

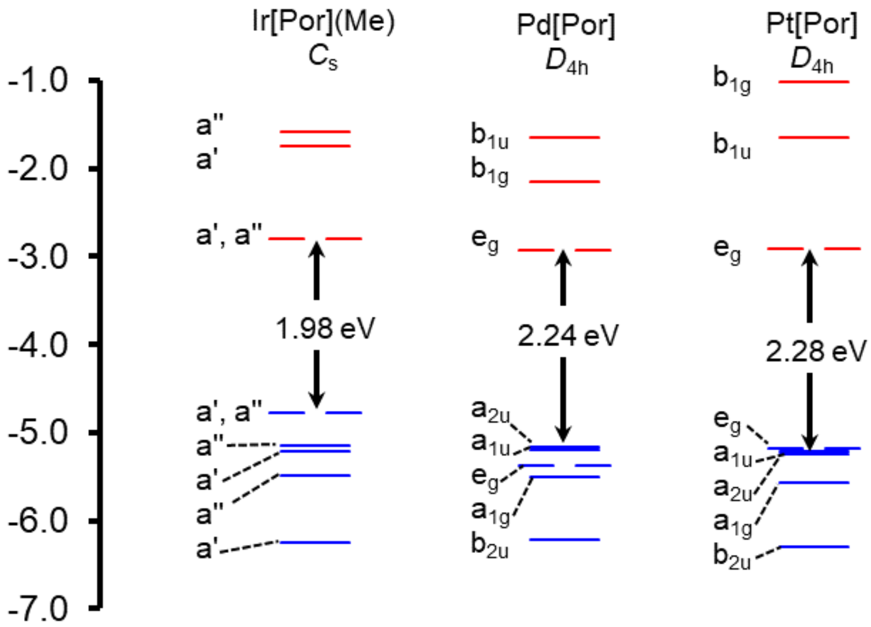

Figure S13. OLYP-D3/STO-ZORA-TZ2P MO energy level diagrams of metalloporphyrins based on ground-state optimized geometries.

D. DFT OLYP-D3/STO-ZORA-TZ2P frontier MOs

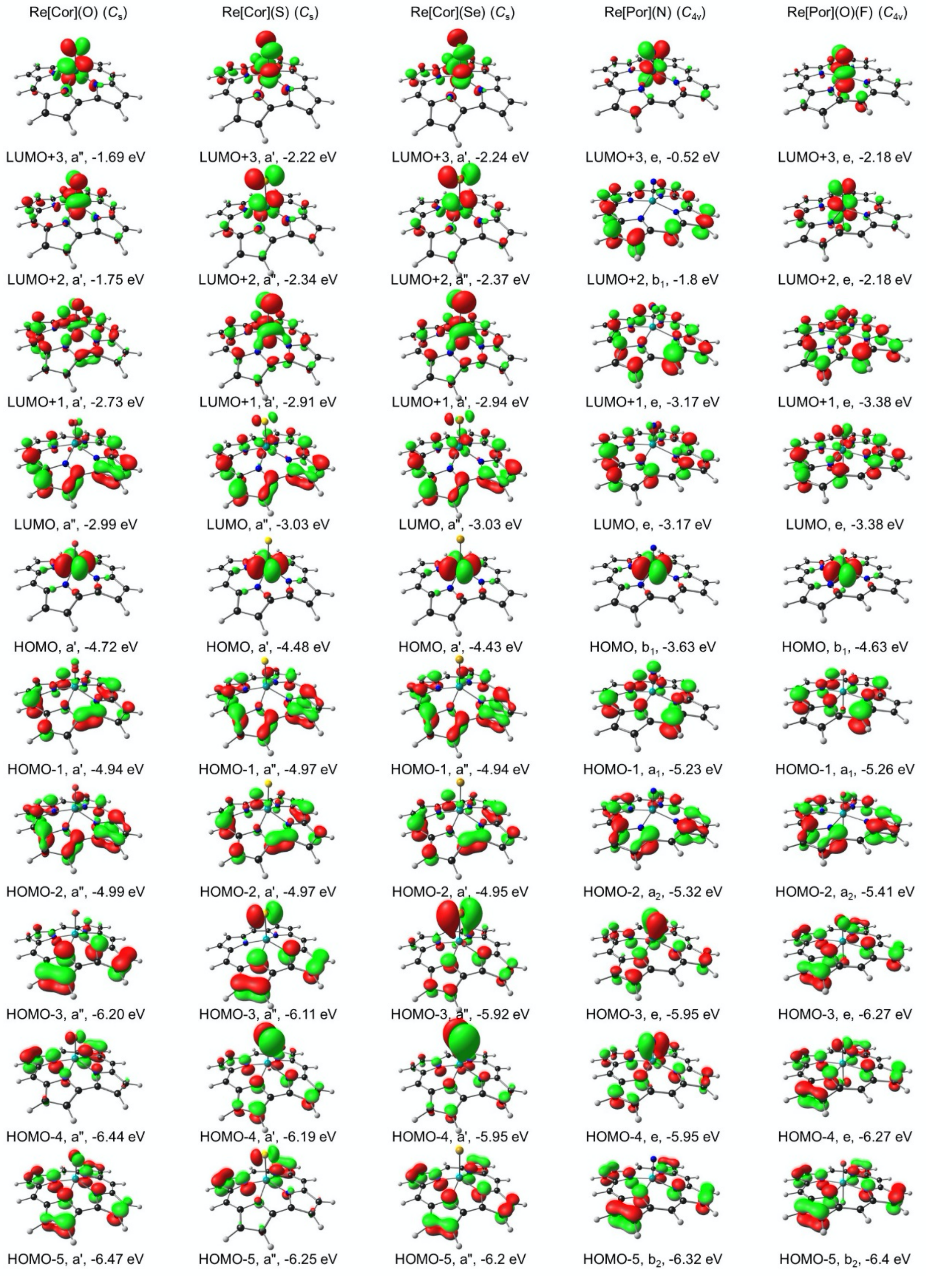

Figure S14. OLYP-D3/STO-ZORA-TZ2P frontier MOs (with a surface isovalue of 0.005 e/Å^3) of rhenium porphyrins and corroles, along with irreps and orbital energies (eV).

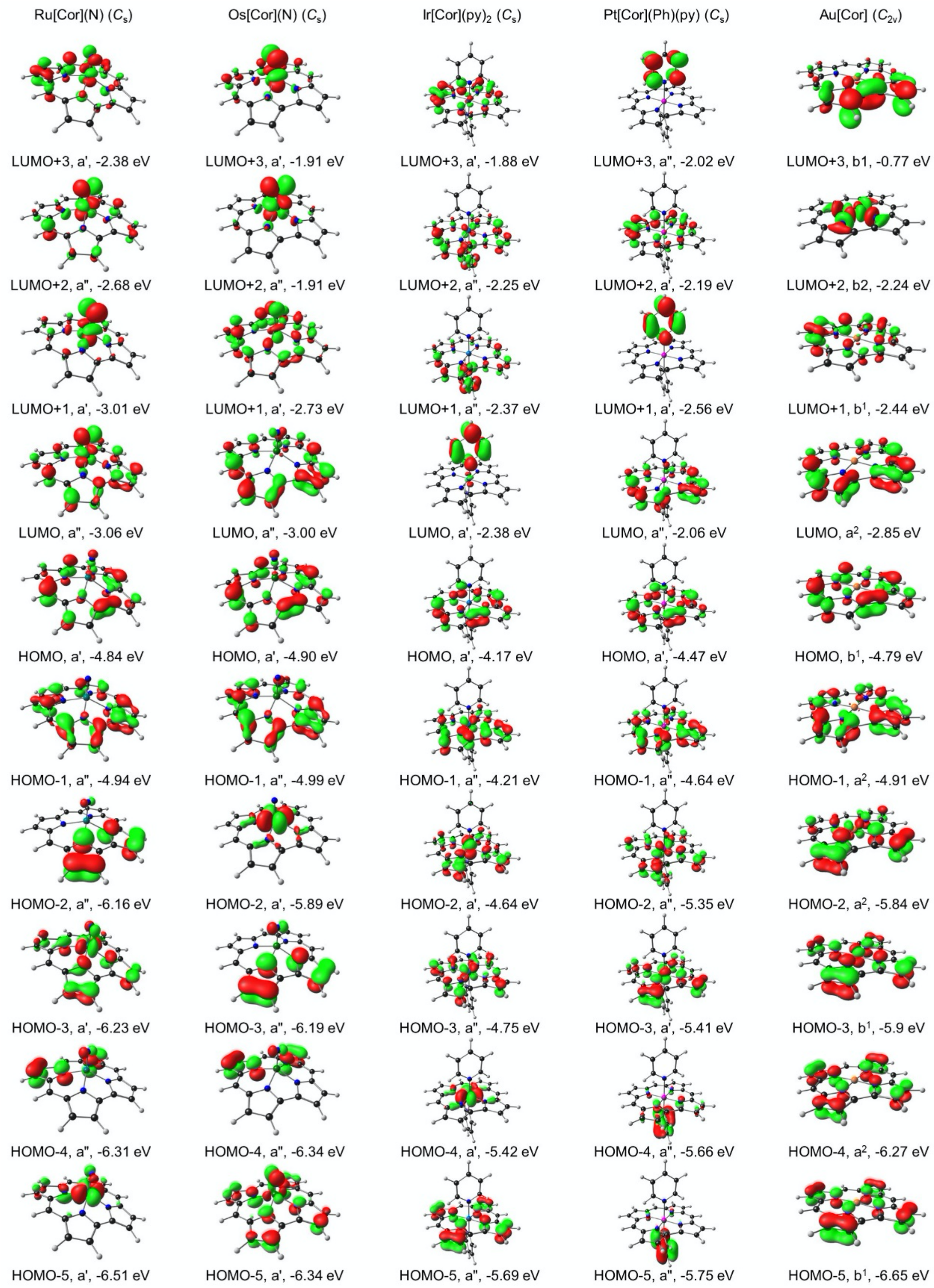

Figure S15. OLYP-D3/STO-ZORA-TZ2P frontier MOs (with a surface isovalue of 0.005 e/Å^3) of metallocorroles, along with irreps and orbital energies (eV).

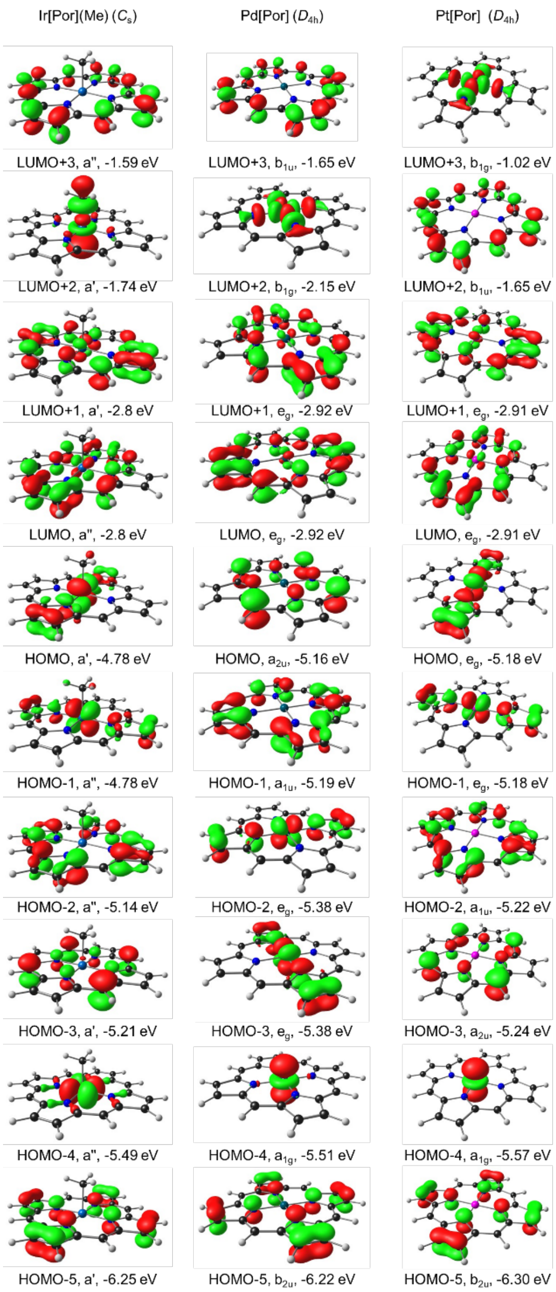

Figure S16. OLYP-D3/STO-ZORA-TZ2P frontier MOs (with a surface isovalue of 0.005 e/Å^3) of metalloporphyrins, along with irreps and orbital energies.

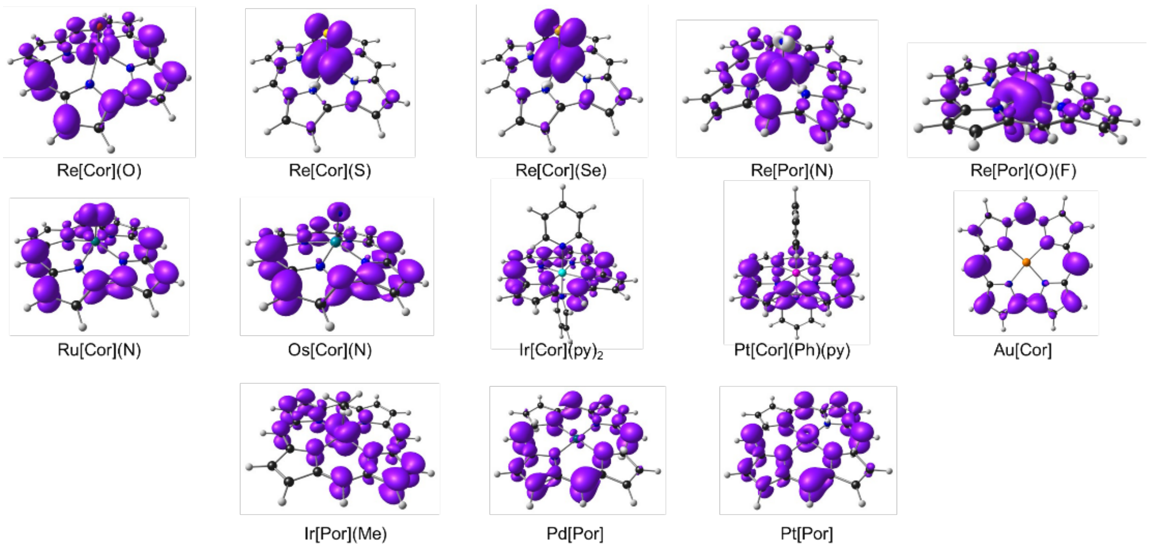

Figure S17. Spin density plots (with a surface isovalue of 0.004 e/Å^3) for OLYP-D3/STO-ZORA-TZ2P triplet optimized structures.

F. Optimized OLYP-D3/STO-ZORA-TZ2P Cartesian coordinates (Å)

Re porphyrins and corroles, $M_S = 0$

1. Re[Cor](O), C_s

Re	-0.243605000	-0.343309000	0.000000000
C	0.007771000	0.506920000	3.338229000
C	0.007771000	0.506920000	-3.338229000
C	1.034640000	-0.248146000	2.771909000
C	1.034640000	-0.248146000	-2.771909000
C	2.140655000	-0.833280000	3.459558000
C	2.140655000	-0.833280000	-3.459558000
C	2.408147000	-1.168082000	1.237108000
C	2.408147000	-1.168082000	-1.237108000
C	2.949201000	-1.508069000	0.000000000
C	2.980277000	-1.391668000	2.525211000
C	2.980277000	-1.391668000	-2.525211000
C	-0.965912000	1.173221000	2.591588000
C	-0.965912000	1.173221000	-2.591588000
C	-1.946031000	2.141831000	2.953926000
C	-1.946031000	2.141831000	-2.953926000
C	-1.961157000	1.920190000	0.704139000
C	-1.961157000	1.920190000	-0.704139000
C	-2.550390000	2.607398000	1.788787000
C	-2.550390000	2.607398000	-1.788787000
H	0.015512000	0.634302000	4.416289000
H	0.015512000	0.634302000	-4.416289000
H	2.274904000	-0.801306000	4.533510000
H	2.274904000	-0.801306000	-4.533510000
H	3.896295000	-2.039095000	0.000000000
H	3.916489000	-1.907234000	2.699043000
H	3.916489000	-1.907234000	-2.699043000
H	-2.151837000	2.466055000	3.966190000
H	-2.151837000	2.466055000	-3.966190000
H	-3.314897000	3.369936000	1.715238000
H	-3.314897000	3.369936000	-1.715238000
N	1.198385000	-0.493708000	1.400357000
N	1.198385000	-0.493708000	-1.400357000
N	-1.026749000	1.024885000	1.219940000
N	-1.026749000	1.024885000	-1.219940000
O	-1.155033000	-1.752166000	0.000000000

2. Re[Cor](S), C_s

Re	-0.225655000	-0.321495000	0.000000000
C	0.015698000	0.519976000	3.336016000
C	0.015698000	0.519976000	-3.336016000
C	1.040615000	-0.241912000	2.774374000
C	1.040615000	-0.241912000	-2.774374000
C	2.143026000	-0.833881000	3.462547000
C	2.143026000	-0.833881000	-3.462547000
C	2.416476000	-1.156217000	1.238505000
C	2.416476000	-1.156217000	-1.238505000
C	2.960583000	-1.485857000	0.000000000
C	2.983581000	-1.389660000	2.527061000
C	2.983581000	-1.389660000	-2.527061000
C	-0.956551000	1.186102000	2.589334000
C	-0.956551000	1.186102000	-2.589334000
C	-1.941282000	2.151039000	2.953985000
C	-1.941282000	2.151039000	-2.953985000
C	-1.952936000	1.934241000	0.703569000
C	-1.952936000	1.934241000	-0.703569000
C	-2.546688000	2.616096000	1.790460000
C	-2.546688000	2.616096000	-1.790460000
H	0.023428000	0.652254000	4.413706000
H	0.023428000	0.652254000	-4.413706000
H	2.275586000	-0.808239000	4.536963000
H	2.275586000	-0.808239000	-4.536963000
H	3.913607000	-2.006412000	0.000000000
H	3.918204000	-1.907611000	2.700961000
H	3.918204000	-1.907611000	-2.700961000
H	-2.150061000	2.471581000	3.966985000
H	-2.150061000	2.471581000	-3.966985000
H	-3.313713000	3.376357000	1.718277000
H	-3.313713000	3.376357000	-1.718277000
N	1.204444000	-0.485017000	1.403341000
N	1.204444000	-0.485017000	-1.403341000
N	-1.020876000	1.036265000	1.217173000
N	-1.020876000	1.036265000	-1.217173000
S	-1.359970000	-2.068996000	0.000000000

3. Re[Cor](Se). C_s

Re	-0.241841000	-0.341739000	0.000000000
C	0.013092000	0.516437000	3.336129000
C	0.013092000	0.516437000	-3.336129000
C	1.036183000	-0.246985000	2.772642000
C	1.036183000	-0.246985000	-2.772642000
C	2.153425000	-0.813579000	3.460547000
C	2.153425000	-0.813579000	-3.460547000
C	2.405213000	-1.168437000	1.238263000
C	2.405213000	-1.168437000	-1.238263000

C	2.949213000	-1.500001000	0.000000000
C	2.990152000	-1.377529000	2.524475000
C	2.990152000	-1.377529000	-2.524475000
C	-0.964215000	1.174963000	2.589240000
C	-0.964215000	1.174963000	-2.589240000
C	-1.935102000	2.155185000	2.953350000
C	-1.935102000	2.155185000	-2.953350000
C	-1.956256000	1.923071000	0.703448000
C	-1.956256000	1.923071000	-0.703448000
C	-2.536216000	2.623762000	1.786999000
C	-2.536216000	2.623762000	-1.786999000
H	0.033799000	0.666279000	4.411395000
H	0.033799000	0.666279000	-4.411395000
H	2.299015000	-0.763590000	4.532485000
H	2.299015000	-0.763590000	-4.532485000
H	3.909493000	-2.007399000	0.000000000
H	3.933935000	-1.879737000	2.695954000
H	3.933935000	-1.879737000	-2.695954000
H	-2.132679000	2.487429000	3.964576000
H	-2.132679000	2.487429000	-3.964576000
H	-3.288872000	3.397690000	1.711016000
H	-3.288872000	3.397690000	-1.711016000
N	1.186890000	-0.510041000	1.403825000
N	1.186890000	-0.510041000	-1.403825000
N	-1.034941000	1.015335000	1.218280000
N	-1.034941000	1.015335000	-1.218280000
Se	-1.457243000	-2.191376000	0.000000000

4. Re[Por](N), C_{4v}

Re	0.000000000	0.000000000	-0.306270000
C	0.000000000	3.435133000	0.219545000
C	0.000000000	-3.435133000	0.219545000
C	1.242321000	2.807157000	0.214933000
C	1.242321000	-2.807157000	0.214933000
C	2.512697000	3.479206000	0.296555000
C	2.512697000	-3.479206000	0.296555000
C	2.807157000	1.242321000	0.214933000
C	2.807157000	-1.242321000	0.214933000
C	3.435133000	0.000000000	0.219545000
C	3.479206000	2.512697000	0.296555000
C	3.479206000	-2.512697000	0.296555000
C	-1.242321000	2.807157000	0.214933000
C	-1.242321000	-2.807157000	0.214933000
C	-2.512697000	3.479206000	0.296555000
C	-2.512697000	-3.479206000	0.296555000
C	-2.807157000	1.242321000	0.214933000
C	-2.807157000	-1.242321000	0.214933000
C	-3.435133000	0.000000000	0.219545000
C	-3.479206000	2.512697000	0.296555000

C	-3.479206000	-2.512697000	0.296555000
H	0.000000000	4.520598000	0.264784000
H	0.000000000	-4.520598000	0.264784000
H	2.637624000	4.553059000	0.355529000
H	2.637624000	-4.553059000	0.355529000
H	4.520598000	0.000000000	0.264784000
H	4.553059000	2.637624000	0.355529000
H	4.553059000	-2.637624000	0.355529000
H	-2.637624000	4.553059000	0.355529000
H	-2.637624000	-4.553059000	0.355529000
H	-4.520598000	0.000000000	0.264784000
H	-4.553059000	2.637624000	0.355529000
H	-4.553059000	-2.637624000	0.355529000
N	0.000000000	0.000000000	-1.954288000
N	1.443522000	1.443522000	0.157059000
N	1.443522000	-1.443522000	0.157059000
N	-1.443522000	1.443522000	0.157059000
N	-1.443522000	-1.443522000	0.157059000

5. Re[Por](O)(F), C_{4v}

Re	0.000000000	0.000000000	-0.170200000
C	0.000000000	-3.442216000	0.051813000
C	1.249706000	2.819796000	0.044612000
C	1.249706000	-2.819796000	0.044612000
C	2.523289000	3.491974000	0.093064000
C	2.523289000	-3.491974000	0.093064000
C	2.819796000	1.249706000	0.044612000
C	2.819796000	-1.249706000	0.044612000
C	3.442216000	0.000000000	0.051813000
C	3.491974000	2.523289000	0.093064000
C	3.491974000	-2.523289000	0.093064000
C	-1.249706000	2.819796000	0.044612000
C	-1.249706000	-2.819796000	0.044612000
C	-2.523289000	3.491974000	0.093064000
C	-2.523289000	-3.491974000	0.093064000
C	-2.819796000	1.249706000	0.044612000
C	-2.819796000	-1.249706000	0.044612000
C	-3.442216000	0.000000000	0.051813000
C	-3.491974000	2.523289000	0.093064000
C	-3.491974000	-2.523289000	0.093064000
F	0.000000000	0.000000000	1.767909000
H	0.000000000	4.528229000	0.085124000
H	0.000000000	-4.528229000	0.085124000
H	2.651815000	4.566497000	0.128662000
H	2.651815000	-4.566497000	0.128662000
H	4.528229000	0.000000000	0.085124000
H	4.566497000	2.651815000	0.128662000
H	4.566497000	-2.651815000	0.128662000
H	-2.651815000	4.566497000	0.128662000

H	-2.651815000	-4.566497000	0.128662000
H	-4.528229000	0.000000000	0.085124000
H	-4.566497000	2.651815000	0.128662000
H	-4.566497000	-2.651815000	0.128662000
N	1.461801000	1.461801000	0.005974000
N	1.461801000	-1.461801000	0.005974000
N	-1.461801000	1.461801000	0.005974000
N	-1.461801000	-1.461801000	0.005974000
O	0.000000000	0.000000000	-1.856922000
C	0.000000000	3.442216000	0.051813000

Other metallocorroles, $M_S = 0$

6. Ru[Cor](N). C_s

Ru	0.086255000	0.544297000	0.000000000
C	0.974402000	-0.306106000	2.778099000
C	0.974402000	-0.306106000	-2.778099000
C	2.185597000	-0.638380000	3.469192000
C	2.185597000	-0.638380000	-3.469192000
C	2.601559000	-0.576327000	1.241157000
C	2.601559000	-0.576327000	-1.241157000
C	3.178000000	-0.807847000	2.532231000
C	3.178000000	-0.807847000	-2.532231000
C	3.228372000	-0.692666000	0.000000000
C	-0.293841000	-0.159190000	3.344501000
C	-0.293841000	-0.159190000	-3.344501000
C	-1.465419000	-0.001334000	2.596216000
C	-1.465419000	-0.001334000	-2.596216000
C	-2.697650000	0.108041000	0.705665000
C	-2.697650000	0.108041000	-0.705665000
C	-2.847242000	-0.071108000	2.956640000
C	-2.847242000	-0.071108000	-2.956640000
C	-3.604384000	-0.012599000	1.788487000
C	-3.604384000	-0.012599000	-1.788487000
H	2.259436000	-0.761708000	4.542517000
H	2.259436000	-0.761708000	-4.542517000
H	4.210668000	-1.085962000	2.701206000
H	4.210668000	-1.085962000	-2.701206000
H	4.282689000	-0.951362000	0.000000000
H	-0.374637000	-0.255652000	4.422754000
H	-0.374637000	-0.255652000	-4.422754000
H	-3.218956000	-0.184893000	3.967242000
H	-3.218956000	-0.184893000	-3.967242000
H	-4.681981000	-0.074128000	1.708081000
H	-4.681981000	-0.074128000	-1.708081000
N	0.374207000	2.129872000	0.000000000
N	1.265659000	-0.259073000	1.416739000

N	1.265659000	-0.259073000	-1.416739000
N	-1.418777000	0.140452000	1.231627000
N	-1.418777000	0.140452000	-1.231627000

7. Os[Cor](N), C_s

Os	0.000914000	0.426622000	0.000000000
C	0.257949000	-0.325055000	3.339777000
C	0.257949000	-0.325055000	-3.339777000
C	1.444208000	-0.351227000	2.597822000
C	1.444208000	-0.351227000	-2.597822000
C	2.687578000	-0.430883000	0.706505000
C	2.687578000	-0.430883000	-0.706505000
C	2.798086000	-0.622385000	2.959359000
C	2.798086000	-0.622385000	-2.959359000
C	3.558494000	-0.679149000	1.794161000
C	3.558494000	-0.679149000	-1.794161000
C	-1.019461000	-0.256487000	2.778497000
C	-1.019461000	-0.256487000	-2.778497000
C	-2.268770000	-0.371032000	3.466633000
C	-2.268770000	-0.371032000	-3.466633000
C	-2.676739000	-0.226641000	1.242318000
C	-2.676739000	-0.226641000	-1.242318000
C	-3.277392000	-0.354758000	2.533262000
C	-3.277392000	-0.354758000	-2.533262000
C	-3.311498000	-0.229195000	0.000000000
H	0.321153000	-0.431162000	4.418317000
H	0.321153000	-0.431162000	-4.418317000
H	3.148954000	-0.784430000	3.970676000
H	3.148954000	-0.784430000	-3.970676000
H	4.615670000	-0.898758000	1.720773000
H	4.615670000	-0.898758000	-1.720773000
H	-2.364515000	-0.480652000	4.539646000
H	-2.364515000	-0.480652000	-4.539646000
H	-4.342483000	-0.440234000	2.707928000
H	-4.342483000	-0.440234000	-2.707928000
H	-4.395379000	-0.292600000	0.000000000
N	1.423139000	-0.205340000	1.230632000
N	1.423139000	-0.205340000	-1.230632000
N	-0.007939000	2.067759000	0.000000000
N	-1.301865000	-0.151348000	1.414705000
N	-1.301865000	-0.151348000	-1.414705000

8. Ir[Cor](py)$_2$, C_s

Ir	0.007021000	-0.009137000	0.000000000
C	0.054406000	0.267384000	3.329131000
C	0.054406000	0.267384000	-3.329131000
C	0.137645000	1.486195000	2.612468000

C	0.137645000	1.486195000	-2.612468000
C	0.196336000	2.758963000	0.720556000
C	0.196336000	2.758963000	-0.720556000
C	0.231144000	2.869320000	3.000529000
C	0.231144000	2.869320000	-3.000529000
C	0.270340000	3.643064000	1.841613000
C	0.270340000	3.643064000	-1.841613000
C	2.603777000	-1.436539000	0.000000000
C	2.844233000	0.880308000	0.000000000
C	3.979801000	-1.611331000	0.000000000
C	4.227381000	0.768211000	0.000000000
C	4.814587000	-0.495059000	0.000000000
C	-0.064892000	-1.035144000	2.795679000
C	-0.064892000	-1.035144000	-2.795679000
C	-0.196215000	-2.285556000	3.506625000
C	-0.196215000	-2.285556000	-3.506625000
C	-0.265692000	-2.702170000	1.267041000
C	-0.265692000	-2.702170000	-1.267041000
C	-0.315512000	-3.297399000	2.582708000
C	-0.315512000	-3.297399000	-2.582708000
C	-0.338784000	-3.319511000	0.000000000
C	-2.712475000	0.271166000	1.164371000
C	-2.712475000	0.271166000	-1.164371000
C	-4.090885000	0.430784000	1.196567000
C	-4.090885000	0.430784000	-1.196567000
C	-4.800650000	0.510651000	0.000000000
H	0.069831000	0.332531000	4.413886000
H	0.069831000	0.332531000	-4.413886000
H	0.266437000	3.235728000	4.018927000
H	0.266437000	3.235728000	-4.018927000
H	0.337485000	4.722649000	1.795392000
H	0.337485000	4.722649000	-1.795392000
H	1.924208000	-2.276494000	0.000000000
H	2.350771000	1.841600000	0.000000000
H	4.382329000	-2.620243000	0.000000000
H	4.828928000	1.672601000	0.000000000
H	5.895729000	-0.607319000	0.000000000
H	-0.195892000	-2.382039000	4.585587000
H	-0.195892000	-2.382039000	-4.585587000
H	-0.431005000	-4.356007000	2.780150000
H	-0.431005000	-4.356007000	-2.780150000
H	-0.457578000	-4.399509000	0.000000000
H	-2.124450000	0.210675000	2.069070000
H	-2.124450000	0.210675000	-2.069070000
H	-4.592376000	0.492235000	2.158115000
H	-4.592376000	0.492235000	-2.158115000
H	-5.880485000	0.635474000	0.000000000
N	0.124842000	1.490275000	1.248116000
N	0.124842000	1.490275000	-1.248116000
N	2.037555000	-0.206754000	0.000000000
N	-0.113294000	-1.352008000	1.450300000

N	-0.113294000	-1.352008000	-1.450300000
N	-2.029471000	0.186234000	0.000000000

9. Pt[Cor](Ph)(py), C_s

Pt	-0.040854000	0.043586000	0.000000000
C	0.199403000	0.190271000	3.332486000
C	0.199403000	0.190271000	-3.332486000
C	1.203874000	0.870415000	2.611356000
C	1.203874000	0.870415000	-2.611356000
C	1.687040000	-2.345828000	1.159743000
C	1.687040000	-2.345828000	-1.159743000
C	2.271276000	1.556489000	0.716774000
C	2.271276000	1.556489000	-0.716774000
C	2.356889000	1.639941000	2.992850000
C	2.356889000	1.639941000	-2.992850000
C	2.516196000	-3.462905000	1.201028000
C	2.516196000	-3.462905000	-1.201028000
C	2.937205000	-4.032601000	0.000000000
C	3.004625000	2.059745000	1.833133000
C	3.004625000	2.059745000	-1.833133000
C	-0.660998000	2.963540000	0.000000000
C	-0.858325000	-0.565038000	2.793614000
C	-0.858325000	-0.565038000	-2.793614000
C	-1.219941000	1.685577000	0.000000000
C	-1.492217000	4.086867000	0.000000000
C	-1.871069000	-1.310164000	3.499749000
C	-1.871069000	-1.310164000	-3.499749000
C	-2.192883000	-1.580308000	1.261129000
C	-2.192883000	-1.580308000	-1.261129000
C	-2.606750000	1.534394000	0.000000000
C	-2.680422000	-1.925094000	2.573777000
C	-2.680422000	-1.925094000	-2.573777000
C	-2.688897000	-1.957153000	0.000000000
C	-2.879221000	3.942830000	0.000000000
C	-3.431197000	2.662065000	0.000000000
H	0.244356000	0.241858000	4.416527000
H	0.244356000	0.241858000	-4.416527000
H	0.412073000	3.100170000	0.000000000
H	1.331960000	-1.858965000	2.060290000
H	1.331960000	-1.858965000	-2.060290000
H	2.657513000	1.850810000	4.011222000
H	2.657513000	1.850810000	-4.011222000
H	2.822977000	-3.871969000	2.159506000
H	2.822977000	-3.871969000	-2.159506000
H	3.585466000	-4.905652000	0.000000000
H	3.906052000	2.656533000	1.780526000
H	3.906052000	2.656533000	-1.780526000
H	-1.044488000	5.078984000	0.000000000
H	-1.953255000	-1.358154000	4.578414000

H	-1.953255000	-1.358154000	-4.578414000
H	-3.056935000	0.550926000	0.000000000
H	-3.523160000	4.819370000	0.000000000
H	-3.535166000	-2.561072000	2.765865000
H	-3.535166000	-2.561072000	-2.765865000
H	-3.561973000	-2.603099000	0.000000000
H	-4.511762000	2.530331000	0.000000000
N	1.215036000	0.859708000	1.249968000
N	1.215036000	0.859708000	-1.249968000
N	1.282683000	-1.806985000	0.000000000
N	-1.103840000	-0.771436000	1.449950000
N	-1.103840000	-0.771436000	-1.449950000

10. Au[Cor], C_{2v}

Au	0.000000000	0.000000000	0.016949000
N	0.000000000	1.446131000	1.349317000
N	0.000000000	-1.446131000	1.349317000
N	0.000000000	-1.249215000	-1.475084000
N	0.000000000	1.249215000	-1.475084000
C	0.000000000	2.793321000	1.030917000
C	0.000000000	3.493484000	2.288520000
C	0.000000000	2.566764000	3.304045000
C	0.000000000	1.256242000	2.710568000
C	0.000000000	0.000000000	3.336179000
C	0.000000000	-1.256242000	2.710568000
C	0.000000000	-2.566764000	3.304045000
C	0.000000000	-3.493484000	2.288520000
C	0.000000000	-2.793321000	1.030917000
C	0.000000000	-3.337550000	-0.263885000
C	0.000000000	-2.612773000	-1.471552000
C	0.000000000	-2.989175000	-2.856566000
C	0.000000000	-1.829959000	-3.629096000
C	0.000000000	-0.714457000	-2.743319000
C	0.000000000	0.714457000	-2.743319000
C	0.000000000	1.829959000	-3.629096000
C	0.000000000	2.989175000	-2.856566000
C	0.000000000	2.612773000	-1.471552000
C	0.000000000	3.337550000	-0.263885000
H	0.000000000	-4.571865000	2.382919000
H	0.000000000	-2.756038000	4.369683000
H	0.000000000	2.756038000	4.369683000
H	0.000000000	4.571865000	2.382919000
H	0.000000000	4.007912000	-3.222177000
H	0.000000000	1.776983000	-4.709892000
H	0.000000000	-1.776983000	-4.709892000
H	0.000000000	-4.007912000	-3.222177000
H	0.000000000	4.421297000	-0.330175000
H	0.000000000	0.000000000	4.421992000
H	0.000000000	-4.421297000	-0.330175000

Other metalloporphyrins, $M_S = 0$

11. Ir[Por](Me), C_s

C	0.118998000	1.976829000	0.000000000
C	0.675832000	-0.116645000	4.229455000
C	0.675832000	-0.116645000	-4.229455000
C	1.100301000	-0.130775000	2.852843000
C	1.100301000	-0.130775000	-2.852843000
C	2.418566000	-0.161098000	2.423777000
C	2.418566000	-0.161098000	-2.423777000
C	2.847994000	-0.186994000	1.105939000
C	2.847994000	-0.186994000	-1.105939000
C	4.223842000	-0.227135000	0.681102000
C	4.223842000	-0.227135000	-0.681102000
C	-0.685786000	-0.071108000	4.229694000
C	-0.685786000	-0.071108000	-4.229694000
C	-1.110272000	-0.055047000	2.853738000
C	-1.110272000	-0.055047000	-2.853738000
C	-2.426530000	0.007264000	2.424585000
C	-2.426530000	0.007264000	-2.424585000
C	-2.854801000	0.016569000	1.105980000
C	-2.854801000	0.016569000	-1.105980000
C	-4.229565000	0.072828000	0.681270000
C	-4.229565000	0.072828000	-0.681270000
H	0.660357000	2.286646000	0.898761000
H	0.660357000	2.286646000	-0.898761000
H	1.350642000	-0.138613000	5.075465000
H	1.350642000	-0.138613000	-5.075465000
H	3.186237000	-0.170153000	3.192425000
H	3.186237000	-0.170153000	-3.192425000
H	5.069397000	-0.253177000	1.356345000
H	5.069397000	-0.253177000	-1.356345000
H	-0.895074000	2.387165000	0.000000000
H	-1.360691000	-0.048797000	5.075585000
H	-1.360691000	-0.048797000	-5.075585000
H	-3.193963000	0.049405000	3.192295000
H	-3.193963000	0.049405000	-3.192295000
H	-5.074969000	0.102845000	1.356469000
H	-5.074969000	0.102845000	-1.356469000
Ir	-0.003031000	-0.054700000	0.000000000
N	2.019037000	-0.175290000	0.000000000
N	-0.005671000	-0.106987000	2.023690000
N	-0.005671000	-0.106987000	-2.023690000
N	-2.026346000	-0.029651000	0.000000000

12. Pd[Por], D_{4h}

Pd	0.000000000	0.000000000	0.000000000
C	0.682019000	4.229436000	0.000000000
C	0.682019000	-4.229436000	0.000000000
C	1.103221000	2.849602000	0.000000000
C	1.103221000	-2.849602000	0.000000000
C	2.424780000	2.424780000	0.000000000
C	2.424780000	-2.424780000	0.000000000
C	2.849602000	1.103221000	0.000000000
C	2.849602000	-1.103221000	0.000000000
C	4.229436000	0.682019000	0.000000000
C	4.229436000	-0.682019000	0.000000000
C	-0.682019000	4.229436000	0.000000000
C	-0.682019000	-4.229436000	0.000000000
C	-1.103221000	2.849602000	0.000000000
C	-1.103221000	-2.849602000	0.000000000
C	-2.424780000	2.424780000	0.000000000
C	-2.424780000	-2.424780000	0.000000000
C	-2.849602000	1.103221000	0.000000000
C	-2.849602000	-1.103221000	0.000000000
C	-4.229436000	0.682019000	0.000000000
C	-4.229436000	-0.682019000	0.000000000
H	1.360102000	5.073230000	0.000000000
H	1.360102000	-5.073230000	0.000000000
H	3.192777000	3.192777000	0.000000000
H	3.192777000	-3.192777000	0.000000000
H	5.073230000	1.360102000	0.000000000
H	5.073230000	-1.360102000	0.000000000
H	-1.360102000	5.073230000	0.000000000
H	-1.360102000	-5.073230000	0.000000000
H	-3.192777000	3.192777000	0.000000000
H	-3.192777000	-3.192777000	0.000000000
H	-5.073230000	1.360102000	0.000000000
H	-5.073230000	-1.360102000	0.000000000
N	0.000000000	2.030101000	0.000000000
N	0.000000000	-2.030101000	0.000000000
N	2.030101000	0.000000000	0.000000000
N	-2.030101000	0.000000000	0.000000000

13. Pt[Por], D_{4h}

Pt	0.000000000	0.000000000	0.000000000
C	0.681258000	4.224251000	0.000000000
C	0.681258000	-4.224251000	0.000000000
C	1.104399000	2.847394000	0.000000000
C	1.104399000	-2.847394000	0.000000000
C	2.422390000	2.422390000	0.000000000
C	2.422390000	-2.422390000	0.000000000
C	2.847394000	1.104399000	0.000000000

C	2.847394000	-1.104399000	0.000000000
C	4.224251000	0.681258000	0.000000000
C	4.224251000	-0.681258000	0.000000000
C	-0.681258000	4.224251000	0.000000000
C	-0.681258000	-4.224251000	0.000000000
C	-1.104399000	2.847394000	0.000000000
C	-1.104399000	-2.847394000	0.000000000
C	-2.422390000	2.422390000	0.000000000
C	-2.422390000	-2.422390000	0.000000000
C	-2.847394000	1.104399000	0.000000000
C	-2.847394000	-1.104399000	0.000000000
C	-4.224251000	0.681258000	0.000000000
C	-4.224251000	-0.681258000	0.000000000
H	1.358107000	5.068774000	0.000000000
H	1.358107000	-5.068774000	0.000000000
H	3.190313000	3.190313000	0.000000000
H	3.190313000	-3.190313000	0.000000000
H	5.068774000	1.358107000	0.000000000
H	5.068774000	-1.358107000	0.000000000
H	-1.358107000	5.068774000	0.000000000
H	-1.358107000	-5.068774000	0.000000000
H	-3.190313000	3.190313000	0.000000000
H	-3.190313000	-3.190313000	0.000000000
H	-5.068774000	1.358107000	0.000000000
H	-5.068774000	-1.358107000	0.000000000
N	0.000000000	2.022193000	0.000000000
N	0.000000000	-2.022193000	0.000000000
N	2.022193000	0.000000000	0.000000000
N	-2.022193000	0.000000000	0.000000000

Re porphyrins and corroles, M_S = 1

14. Re[Cor](O), C_1

Re	-0.268425000	-0.815375000	0.094489000
C	0.038864000	0.170761000	-3.168294000
C	0.702365000	-0.225236000	2.805107000
C	1.193776000	-0.306943000	-2.549552000
C	1.792386000	-0.361585000	3.667216000
C	2.433766000	-0.986018000	1.563542000
C	2.451942000	-0.583821000	-3.142914000
C	2.579941000	-1.028877000	-0.920777000
C	2.873636000	-0.853091000	2.901903000
C	3.115556000	-1.252202000	0.366430000
C	3.299926000	-1.044075000	-2.150700000
C	-0.668478000	0.229400000	2.812435000
C	-1.169269000	0.466816000	-2.537711000
C	-1.616634000	0.766868000	3.685636000
C	-2.353941000	0.987384000	-3.118468000

C	-2.524931000	0.652636000	1.590815000
C	-2.696027000	0.705692000	-0.892108000
C	-2.785763000	1.017257000	2.932990000
C	-3.244568000	0.844367000	0.401559000
C	-3.299295000	1.117499000	-2.116138000
H	0.088954000	0.339744000	-4.240725000
H	1.802660000	-0.128438000	4.723749000
H	2.681278000	-0.439804000	-4.191450000
H	3.876755000	-1.057110000	3.253112000
H	4.167003000	-1.515832000	0.420290000
H	4.340328000	-1.325515000	-2.248901000
H	-1.475427000	0.956955000	4.741417000
H	-2.465310000	1.238194000	-4.166101000
H	-3.709964000	1.448586000	3.294983000
H	-4.247141000	1.254846000	0.466949000
H	-4.305982000	1.504995000	-2.203838000
N	1.090092000	-0.638899000	1.538393000
N	1.274822000	-0.614480000	-1.183621000
N	-1.238659000	0.132500000	1.551031000
N	-1.402029000	0.266812000	-1.169426000
O	-0.796555000	-2.406237000	0.021613000

15. Re[Cor](S), C_1

Re	-0.239969000	-0.747877000	0.055367000
C	0.015811000	0.034574000	-3.180686000
C	0.632520000	-0.288409000	2.804719000
C	1.188802000	-0.434583000	-2.591656000
C	1.731760000	-0.471687000	3.670862000
C	2.432002000	-0.879939000	1.528458000
C	2.484617000	-0.613614000	-3.183734000
C	2.642948000	-0.980259000	-0.943605000
C	2.830615000	-0.837192000	2.892490000
C	3.154860000	-1.102383000	0.348682000
C	3.363458000	-0.947146000	-2.184118000
C	-0.728691000	0.095377000	2.835032000
C	-1.170029000	0.402075000	-2.530273000
C	-1.650674000	0.722403000	3.719767000
C	-2.365126000	0.911610000	-3.111266000
C	-2.530997000	0.680356000	1.615178000
C	-2.661490000	0.840336000	-0.854770000
C	-2.766056000	1.075018000	2.976332000
C	-3.199490000	0.990632000	0.429316000
C	-3.264125000	1.177220000	-2.099819000
H	0.035866000	0.187066000	-4.255767000
H	1.714885000	-0.344635000	4.745253000
H	2.704219000	-0.464484000	-4.233233000
H	3.830696000	-1.051323000	3.247842000
H	4.217087000	-1.307751000	0.442734000
H	4.428846000	-1.117820000	-2.274365000

H	-1.481640000	0.920024000	4.770513000
H	-2.508054000	1.062374000	-4.173758000
H	-3.639843000	1.609737000	3.325399000
H	-4.178490000	1.455870000	0.496019000
H	-4.263566000	1.581275000	-2.200508000
N	1.080061000	-0.549719000	1.505566000
N	1.301162000	-0.722538000	-1.233647000
N	-1.319156000	0.024895000	1.587518000
N	-1.367525000	0.360220000	-1.138700000
S	-0.919643000	-2.751928000	0.025792000

16. Re[Cor](Se), *C_1*

Re	-0.244219000	-0.752057000	0.054691000
C	0.015018000	0.030878000	-3.180032000
C	0.631054000	-0.294282000	2.802164000
C	1.184496000	-0.449048000	-2.591898000
C	1.732731000	-0.465945000	3.668097000
C	2.429871000	-0.887896000	1.527268000
C	2.479076000	-0.628635000	-3.185630000
C	2.638767000	-0.995578000	-0.945258000
C	2.831122000	-0.833863000	2.891045000
C	3.152028000	-1.112154000	0.347809000
C	3.359069000	-0.961468000	-2.186139000
C	-0.728170000	0.093759000	2.832505000
C	-1.167962000	0.407542000	-2.530567000
C	-1.647520000	0.724201000	3.718458000
C	-2.352789000	0.942487000	-3.110499000
C	-2.528296000	0.685112000	1.614031000
C	-2.656380000	0.854418000	-0.855596000
C	-2.761862000	1.079957000	2.975967000
C	-3.193354000	1.005163000	0.428780000
C	-3.250557000	1.212342000	-2.098826000
H	0.038769000	0.189654000	-4.254148000
H	1.717459000	-0.327772000	4.741134000
H	2.697833000	-0.476165000	-4.234699000
H	3.833152000	-1.038966000	3.246038000
H	4.215546000	-1.310786000	0.441611000
H	4.425279000	-1.126818000	-2.276720000
H	-1.476968000	0.920864000	4.768934000
H	-2.490523000	1.105536000	-4.171885000
H	-3.634502000	1.616467000	3.325320000
H	-4.167397000	1.480394000	0.496967000
H	-4.242991000	1.633578000	-2.198162000
N	1.076342000	-0.566355000	1.504964000
N	1.296773000	-0.738657000	-1.234127000
N	-1.319757000	0.024405000	1.585978000
N	-1.370741000	0.354960000	-1.140722000
Se	-0.984747000	-2.877498000	0.056083000

17. Re[Por](N), C_1

Re	-0.011288000	0.003862000	-0.503144000
C	0.002627000	3.430675000	-0.294583000
C	1.209595000	-2.810311000	-0.240960000
C	1.254933000	2.800435000	-0.185435000
C	2.480072000	-3.482170000	-0.154079000
C	2.508754000	3.451618000	-0.074560000
C	2.783375000	-1.253700000	0.100269000
C	2.800443000	1.223053000	0.136311000
C	3.411305000	-0.035794000	0.218676000
C	3.432711000	-2.535717000	0.076727000
C	3.463294000	2.473424000	0.141703000
C	-0.020194000	-3.426314000	-0.341440000
C	-1.224097000	2.813230000	-0.168530000
C	-1.268965000	-2.797643000	-0.190501000
C	-2.491731000	3.483552000	-0.038853000
C	-2.519593000	-3.450342000	-0.054578000
C	-2.787991000	1.251964000	0.195738000
C	-2.804563000	-1.225003000	0.195189000
C	-3.412538000	0.032336000	0.313424000
C	-3.437772000	2.533939000	0.205827000
C	-3.467669000	-2.475184000	0.200207000
H	0.006096000	4.513050000	-0.373691000
H	2.604404000	-4.554724000	-0.231303000
H	2.649787000	4.523233000	-0.127678000
H	4.490033000	-0.046280000	0.341796000
H	4.497047000	-2.676545000	0.216534000
H	4.529311000	2.600188000	0.279429000
H	-0.025942000	-4.507633000	-0.433598000
H	-2.618177000	4.556889000	-0.099974000
H	-2.662382000	-4.521162000	-0.117962000
H	-4.487630000	0.040754000	0.465252000
H	-4.498047000	2.672634000	0.375531000
H	-4.529654000	-2.603705000	0.364994000
N	1.412591000	-1.441751000	-0.094538000
N	1.435768000	1.429854000	-0.059093000
N	-0.033661000	0.016320000	-2.151747000
N	-1.423068000	1.442922000	-0.034144000
N	-1.445853000	-1.428890000	-0.040632000

18. Re[Por](O)(F), C_1

Re	-0.008697000	0.005484000	-0.205283000
C	0.004832000	3.431235000	-0.290944000
C	1.217900000	-2.813905000	-0.240297000
C	1.260399000	2.809111000	-0.202476000
C	2.489280000	-3.491671000	-0.149810000
C	2.525541000	3.463654000	-0.130399000

C	2.787314000	-1.259964000	0.096773000
C	2.808352000	1.234216000	0.119878000
C	3.411237000	-0.028339000	0.209522000
C	3.443548000	-2.544765000	0.073024000
C	3.479338000	2.491852000	0.084599000
C	-0.024217000	-3.422007000	-0.327846000
C	-1.234799000	2.822133000	-0.178254000
C	-1.276200000	-2.799644000	-0.204870000
C	-2.503014000	3.499227000	-0.048302000
C	-2.540588000	-3.453298000	-0.119348000
C	-2.795982000	1.265473000	0.185199000
C	-2.816130000	-1.228141000	0.172626000
C	-3.417087000	0.032483000	0.296161000
C	-3.451447000	2.550833000	0.192775000
C	-3.488649000	-2.484168000	0.131494000
F	0.022452000	-0.001320000	1.754737000
H	0.009310000	4.514888000	-0.363615000
H	2.614670000	-4.564063000	-0.225699000
H	2.670678000	4.532895000	-0.213270000
H	4.491140000	-0.038793000	0.325231000
H	4.508551000	-2.686418000	0.205305000
H	4.548313000	2.621080000	0.194037000
H	-0.031292000	-4.504592000	-0.414038000
H	-2.629649000	4.572457000	-0.109606000
H	-2.689076000	-4.520629000	-0.219133000
H	-4.494153000	0.040412000	0.436099000
H	-4.512321000	2.691711000	0.355496000
H	-4.555522000	-2.614057000	0.259740000
N	1.429585000	-1.452227000	-0.103191000
N	1.454078000	1.449706000	-0.055687000
N	-1.444130000	1.458993000	-0.048801000
N	-1.465084000	-1.441457000	-0.034058000
O	-0.026726000	0.021422000	-1.924527000

Other metallocorroles, $M_S = 1$

19. Ru[Cor](N), C_1

Ru	-0.235165000	-0.719676000	0.091750000
C	0.683843000	-0.292907000	2.812086000
C	1.178985000	-0.391052000	-2.566553000
C	1.765478000	-0.453452000	3.694502000
C	2.448539000	-0.939930000	1.566301000
C	2.452001000	-0.620166000	-3.168114000
C	2.609462000	-0.956304000	-0.927176000
C	2.869019000	-0.866995000	2.923799000
C	3.144202000	-1.161400000	0.367204000
C	3.330991000	-0.974751000	-2.164222000

C	-0.002582000	0.042272000	-3.181182000
C	-0.698524000	0.162734000	2.819624000
C	-1.208179000	0.381646000	-2.553089000
C	-1.642705000	0.704286000	3.709356000
C	-2.380744000	0.935478000	-3.142599000
C	-2.516871000	0.688969000	1.595672000
C	-2.679530000	0.770082000	-0.896615000
C	-2.785254000	1.025773000	2.954856000
C	-3.220389000	0.922655000	0.406192000
C	-3.287974000	1.173213000	-2.126649000
H	0.035840000	0.180490000	-4.258644000
H	1.748406000	-0.285258000	4.763150000
H	2.663339000	-0.517440000	-4.225140000
H	3.874464000	-1.054446000	3.278181000
H	4.196557000	-1.422351000	0.425508000
H	4.386570000	-1.195662000	-2.256371000
H	-1.505799000	0.850822000	4.772723000
H	-2.503871000	1.138262000	-4.199104000
H	-3.690328000	1.497203000	3.314927000
H	-4.218015000	1.345325000	0.473885000
H	-4.271894000	1.616479000	-2.209043000
N	1.112284000	-0.609154000	1.548559000
N	1.300435000	-0.620737000	-1.204582000
N	-0.739319000	-2.257437000	0.013235000
N	-1.253394000	0.143379000	1.566049000
N	-1.424226000	0.277826000	-1.185533000

20. Os[Cor](N), *C*$_1$

Os	-0.242600000	-0.738648000	0.096783000
C	0.002390000	0.058093000	-3.183571000
C	0.684472000	-0.292423000	2.820338000
C	1.176987000	-0.386605000	-2.568671000
C	1.769117000	-0.427148000	3.691331000
C	2.441764000	-0.631398000	-3.166376000
C	2.450401000	-0.942516000	1.567052000
C	2.606904000	-0.964717000	-0.924966000
C	2.875171000	-0.844494000	2.916927000
C	3.144956000	-1.174225000	0.368166000
C	3.321414000	-0.990847000	-2.160225000
C	-0.696343000	0.161329000	2.827899000
C	-1.200568000	0.395776000	-2.555854000
C	-1.643172000	0.688289000	3.710835000
C	-2.369829000	0.950455000	-3.140558000
C	-2.515962000	0.689449000	1.594116000
C	-2.677850000	0.775876000	-0.896168000
C	-2.788520000	1.010915000	2.948595000
C	-3.223243000	0.920683000	0.402805000
C	-3.280349000	1.182740000	-2.124529000

H	0.042648000	0.198446000	-4.260687000
H	1.761200000	-0.233398000	4.755554000
H	2.653771000	-0.530980000	-4.223491000
H	3.884262000	-1.016364000	3.268090000
H	4.197327000	-1.433640000	0.424977000
H	4.374286000	-1.222245000	-2.254896000
H	-1.512956000	0.832905000	4.775009000
H	-2.491604000	1.159206000	-4.196082000
H	-3.700842000	1.466379000	3.311068000
H	-4.223961000	1.335243000	0.470742000
H	-4.265812000	1.621992000	-2.208359000
N	1.106745000	-0.626902000	1.548702000
N	1.295524000	-0.612450000	-1.197072000
N	-0.757793000	-2.292254000	0.016854000
N	-1.245761000	0.150176000	1.560987000
N	-1.416524000	0.281071000	-1.182387000

21. Ir[Cor](py)$_2$, C_1

Ir	-0.027065000	-0.013905000	-0.001422000
C	0.116327000	1.440979000	2.641508000
C	0.145595000	1.523333000	-2.595639000
C	0.285715000	2.799087000	3.055152000
C	0.344140000	2.890706000	-2.964690000
C	0.353258000	2.723636000	0.782308000
C	0.362688000	2.746306000	-0.694430000
C	0.428350000	3.590966000	1.894714000
C	0.474413000	3.645106000	-1.778435000
C	2.690343000	-0.265754000	1.163373000
C	2.694922000	-0.243121000	-1.163415000
C	4.074253000	-0.364153000	1.198064000
C	4.078815000	-0.341322000	-1.194866000
C	4.788738000	-0.405636000	0.002426000
C	-0.025431000	0.316509000	-3.329032000
C	-0.050472000	0.209592000	3.334367000
C	-0.166040000	-1.009211000	-2.806797000
C	-0.173973000	-1.099999000	2.769008000
C	-0.266304000	-2.750177000	1.224488000
C	-0.267963000	-2.708391000	-1.316905000
C	-0.275394000	-2.359042000	3.462215000
C	-0.276095000	-2.244323000	-3.540720000
C	-0.301456000	-3.337228000	-0.056248000
C	-0.331886000	-3.365892000	2.516159000
C	-0.338673000	-3.281024000	-2.627930000
C	-2.621332000	1.435295000	0.038334000
C	-2.849686000	-0.880249000	-0.031776000
C	-3.997941000	1.601616000	0.044192000
C	-4.231907000	-0.777522000	-0.029113000
C	-4.828511000	0.482261000	0.010421000
H	0.302743000	3.149967000	4.079008000

H	0.384263000	3.272263000	-3.976969000
H	0.572523000	4.662777000	1.863037000
H	0.633612000	4.713246000	-1.711738000
H	2.100123000	-0.221566000	2.067470000
H	2.108553000	-0.180364000	-2.068835000
H	4.576202000	-0.403540000	2.160401000
H	4.584421000	-0.361902000	-2.155900000
H	-0.030910000	0.394476000	-4.412548000
H	-0.069215000	0.253260000	4.419675000
H	-0.299718000	-2.469851000	4.538988000
H	-0.300682000	-2.319976000	-4.620500000
H	-0.378239000	-4.422300000	-0.074028000
H	-0.410194000	-4.430951000	2.694788000
H	-0.423357000	-4.339274000	-2.840969000
H	-1.948982000	2.279800000	0.063960000
H	-2.352703000	-1.838453000	-0.060144000
H	-4.404957000	2.608100000	0.075496000
H	-4.826827000	-1.685960000	-0.056817000
H	-5.909980000	0.588655000	0.014628000
N	0.160941000	1.460692000	1.276880000
N	0.162812000	1.501674000	-1.230381000
N	2.006625000	-0.213146000	-0.001069000
N	-0.171966000	-1.336688000	-1.476548000
N	-0.176181000	-1.384306000	1.428739000
N	-2.046203000	0.210018000	0.001386000
H	5.873076000	-0.481088000	0.003691000

22. Pt[Cor](Ph)(py), C_1

Pt	-0.098924000	-0.002064000	-0.000764000
C	0.109486000	1.436891000	2.644260000
C	0.124130000	1.520777000	-2.597549000
C	0.311916000	2.790483000	3.050797000
C	0.312216000	2.721929000	0.778546000
C	0.316814000	2.745251000	-0.690279000
C	0.335107000	2.885767000	-2.959250000
C	0.433638000	3.582396000	1.889740000
C	0.450522000	3.639930000	-1.772481000
C	2.805152000	-0.316765000	1.158989000
C	2.813564000	-0.221652000	-1.160479000
C	4.191693000	-0.418854000	1.200205000
C	4.200320000	-0.320977000	-1.199649000
C	4.902100000	-0.423678000	0.000670000
C	-0.027165000	0.319946000	-3.335209000
C	-0.047026000	0.213518000	3.342373000
C	-0.166833000	-0.999189000	-2.807015000
C	-0.184786000	-1.088165000	2.772046000
C	-0.248662000	-2.236909000	-3.535208000
C	-0.275646000	-2.348246000	3.459737000
C	-0.293268000	-2.693861000	-1.310658000

C	-0.301936000	-2.734384000	1.221370000
C	-0.327249000	-3.269606000	-2.618749000
C	-0.334061000	-3.322986000	-0.054562000
C	-0.348798000	-3.351105000	2.510114000
C	-2.114099000	0.214487000	0.001208000
C	-2.695043000	1.482542000	0.011657000
C	-2.926595000	-0.919392000	-0.007658000
C	-4.086440000	1.611766000	0.015266000
C	-4.317130000	-0.783212000	-0.003789000
C	-4.903420000	0.481930000	0.007831000
H	0.362424000	3.136072000	4.075080000
H	0.395658000	3.263401000	-3.971628000
H	0.596594000	4.651089000	1.853560000
H	0.618247000	4.706224000	-1.701371000
H	2.202776000	-0.300257000	2.059317000
H	2.217947000	-0.131611000	-2.061002000
H	4.698386000	-0.490439000	2.158412000
H	4.713934000	-0.313696000	-2.156762000
H	5.986761000	-0.501018000	0.001459000
H	-0.018659000	0.396610000	-4.418082000
H	-0.048026000	0.256173000	4.427164000
H	-0.245576000	-2.315249000	-4.614682000
H	-0.282385000	-2.460936000	4.536195000
H	-0.396817000	-4.329652000	-2.827104000
H	-0.406072000	-4.407818000	-0.072231000
H	-0.422623000	-4.417100000	2.683569000
H	-2.083720000	2.374502000	0.016900000
H	-2.494427000	-1.910446000	-0.017814000
H	-4.527209000	2.606794000	0.023893000
H	-4.939204000	-1.676295000	-0.010483000
H	-5.985978000	0.586346000	0.010675000
N	0.117948000	1.462407000	1.278513000
N	0.121830000	1.502879000	-1.231604000
N	2.137991000	-0.225551000	-0.001358000
N	-0.199916000	-1.324057000	-1.478442000
N	-0.206253000	-1.370934000	1.433325000

23. Au[Cor], C_1

Au	0.008823000	0.011349000	0.104204000
C	0.713771000	-0.237683000	2.863777000
C	1.197459000	-0.371389000	-2.615186000
C	1.754948000	-0.584761000	3.742226000
C	2.425933000	-0.774941000	-3.217790000
C	2.506509000	-0.813462000	1.580743000
C	2.657187000	-0.853039000	-0.947721000
C	2.870716000	-0.942523000	2.953164000
C	3.190294000	-1.032184000	0.362087000
C	3.319178000	-1.069570000	-2.202191000
C	-0.004287000	0.025746000	-3.223009000

C	-0.680996000	0.215176000	2.871181000
C	-1.201080000	0.418251000	-2.602458000
C	-1.720638000	0.537134000	3.760656000
C	-2.434216000	0.827400000	-3.191857000
C	-2.481720000	0.808989000	1.607430000
C	-2.648012000	0.883928000	-0.919438000
C	-2.841236000	0.906781000	2.983781000
C	-3.173125000	1.044088000	0.396054000
C	-3.319629000	1.112707000	-2.166782000
H	1.704523000	-0.577943000	4.822524000
H	2.599292000	-0.831338000	-4.284630000
H	3.839657000	-1.262950000	3.312402000
H	4.219601000	-1.371442000	0.416977000
H	4.342336000	-1.407159000	-2.302702000
H	-0.008656000	0.029555000	-4.310087000
H	-1.665296000	0.505688000	4.840308000
H	-2.615809000	0.892768000	-4.256811000
H	-3.810743000	1.213757000	3.353081000
H	-4.203912000	1.376972000	0.461576000
H	-4.343750000	1.450168000	-2.256722000
N	1.207298000	-0.387552000	1.591731000
N	1.385635000	-0.434829000	-1.244612000
N	-1.179425000	0.392507000	1.604570000
N	-1.378479000	0.469490000	-1.229919000

Other metalloporphyrins, $M_S = 1$

24. Ir[Por](Me), C_1

Ir	-0.071683000	-0.052543000	0.062535000
C	1.119970000	-2.866808000	-0.097873000
C	1.206612000	2.752997000	0.105080000
C	2.358951000	-3.526310000	-0.126044000
C	2.492900000	3.412056000	0.151984000
C	2.720269000	-1.295654000	-0.055056000
C	2.759620000	1.170819000	0.064104000
C	3.357016000	-2.546540000	-0.101660000
C	3.372939000	-0.056705000	0.014641000
C	3.443942000	2.443248000	0.125301000
C	-0.010008000	3.390201000	0.096851000
C	-0.063260000	-0.053450000	2.101271000
C	-0.133105000	-3.495080000	-0.077543000
C	-1.262059000	2.764048000	0.024554000
C	-1.348530000	-2.857268000	-0.050333000
C	-2.501344000	3.425606000	0.005298000
C	-2.635246000	-3.516509000	-0.055074000
C	-2.864439000	1.195156000	-0.048178000
C	-2.901411000	-1.273576000	-0.039824000
C	-3.499735000	2.447717000	-0.042516000
C	-3.516057000	-0.045628000	-0.048182000

C	-3.585813000	-2.547001000	-0.049108000
H	0.918227000	-0.414167000	2.418061000
H	2.482431000	-4.601166000	-0.149891000
H	2.622312000	4.486048000	0.190570000
H	4.429546000	-2.689846000	-0.104189000
H	4.458130000	-0.077140000	0.025496000
H	4.520844000	2.551903000	0.138303000
H	-0.009221000	4.474839000	0.139263000
H	-0.134974000	-4.580446000	-0.092118000
H	-0.213143000	0.978839000	2.426022000
H	-0.864484000	-0.708837000	2.450476000
H	-2.624119000	4.500342000	0.032501000
H	-2.765119000	-4.591081000	-0.069766000
H	-4.572009000	2.592520000	-0.059116000
H	-4.601332000	-0.026727000	-0.054968000
H	-4.662808000	-2.655457000	-0.057518000
N	1.327929000	-1.477222000	-0.066270000
N	1.394333000	1.385104000	0.047532000
N	-1.470613000	1.375702000	-0.020059000
N	-1.534960000	-1.486699000	-0.042360000

25. Pd[Por], C_1

Pd	-0.005403000	0.000394000	-0.001833000
C	0.008355000	3.434886000	0.026401000
C	1.224943000	-2.810710000	-0.045416000
C	1.259200000	2.804807000	0.010000000
C	2.494235000	-3.499978000	-0.075806000
C	2.518318000	3.468583000	0.005048000
C	2.800084000	-1.263155000	-0.055206000
C	2.803273000	1.236035000	-0.025449000
C	3.431546000	-0.040307000	-0.049358000
C	3.466068000	-2.546204000	-0.082534000
C	3.488569000	2.481738000	-0.017454000
C	-0.018823000	-3.434783000	-0.025047000
C	-1.235482000	2.810533000	0.030429000
C	-1.269541000	-2.804001000	0.002186000
C	-2.504967000	3.501026000	0.045539000
C	-2.528826000	-3.467081000	0.021917000
C	-2.811447000	1.264258000	0.039870000
C	-2.814078000	-1.234683000	0.031034000
C	-3.442687000	0.041667000	0.042744000
C	-3.477336000	2.547648000	0.049339000
C	-3.499173000	-2.480201000	0.040682000
H	0.010000000	4.521259000	0.036542000
H	2.603791000	-4.576608000	-0.088963000
H	2.650031000	4.542551000	0.016387000
H	4.517479000	-0.044099000	-0.063921000
H	4.540500000	-2.675102000	-0.102551000
H	4.564558000	2.595074000	-0.026247000

H	-0.020629000	-4.521042000	-0.031940000
H	-2.613422000	4.578099000	0.049878000
H	-2.660447000	-4.541094000	0.023210000
H	-4.528782000	0.045838000	0.054855000
H	-4.551846000	2.677697000	0.059821000
H	-4.574981000	-2.593496000	0.061655000
N	1.432785000	-1.458351000	-0.035201000
N	1.451059000	1.435970000	-0.008059000
N	-1.443930000	1.458741000	0.026074000
N	-1.461662000	-1.434851000	0.007953000

26. Pt[Por], C_1

Pt	-0.006749000	0.002941000	-0.002195000
C	0.001175000	3.437674000	0.026065000
C	1.222190000	-2.805379000	-0.039904000
C	1.250418000	2.797068000	0.003878000
C	2.490662000	-3.495306000	-0.062137000
C	2.503506000	3.457820000	-0.005988000
C	2.801529000	-1.259405000	-0.052261000
C	2.803555000	1.228943000	-0.031981000
C	3.434152000	-0.045518000	-0.050456000
C	3.462856000	-2.544563000	-0.072547000
C	3.483852000	2.462131000	-0.028831000
C	-0.019030000	-3.430570000	-0.024128000
C	-1.234207000	2.814157000	0.035454000
C	-1.259653000	-2.801159000	-0.001828000
C	-2.505427000	3.498139000	0.055957000
C	-2.520630000	-3.455656000	0.011506000
C	-2.808569000	1.262540000	0.042380000
C	-2.810151000	-1.221253000	0.027833000
C	-3.438256000	0.040339000	0.044240000
C	-3.474383000	2.542817000	0.061288000
C	-3.486375000	-2.473886000	0.028964000
H	0.007731000	4.523412000	0.037767000
H	2.596833000	-4.572587000	-0.068573000
H	2.636803000	4.531527000	0.003308000
H	4.519698000	-0.048017000	-0.064573000
H	4.537236000	-2.674498000	-0.088726000
H	4.559060000	2.578883000	-0.041287000
H	-0.021880000	-4.516632000	-0.029322000
H	-2.616224000	4.574730000	0.066188000
H	-2.651706000	-4.529619000	0.005670000
H	-4.524263000	0.042463000	0.058726000
H	-4.549275000	2.668638000	0.075976000
H	-4.562389000	-2.587790000	0.040154000
N	1.427321000	-1.453110000	-0.036160000
N	1.439983000	1.431338000	-0.010689000
N	-1.440781000	1.455391000	0.024663000
N	-1.447277000	-1.414937000	0.008153000
